# Supplementary material for: Spatial profiling of microbial communities by sequential FISH with error-robust encoding
Source: Nat Commun. 2023 Mar 17;14:1477. doi: 10.1038/s41467-023-37188-3 (PMC10023729; doi:10.1038/s41467-023-37188-3)
Supplement: Supplementary file 1 — Supplementary information [file 41467_2023_37188_MOESM1_ESM.pdf]

Supplementary Information

Spatial profiling of microbial communities by sequential FISH with error-robust encoding

Zhaohui Cao[1,2], Wenlong Zuo[1], Lanxiang Wang[1], Junyu Chen[1], Zepeng Qu[1], Fan Jin[1,2], Lei Dai[1,2*]

[1] CAS Key Laboratory of Quantitative Engineering Biology, Shenzhen Institute of Synthetic Biology, Shenzhen Institutes of Advanced Technology, Chinese Academy of Sciences, Shenzhen 518055, China

[2] University of Chinese Academy of Sciences, Beijing 100049, China

These authors contributed equally: Zhaohui Cao, Wenlong Zuo.

*email: lei.dai@siat.ac.cn

This PDF file includes:

Supplementary Fig. 1 to 19
Supplementary Table 1 to 5

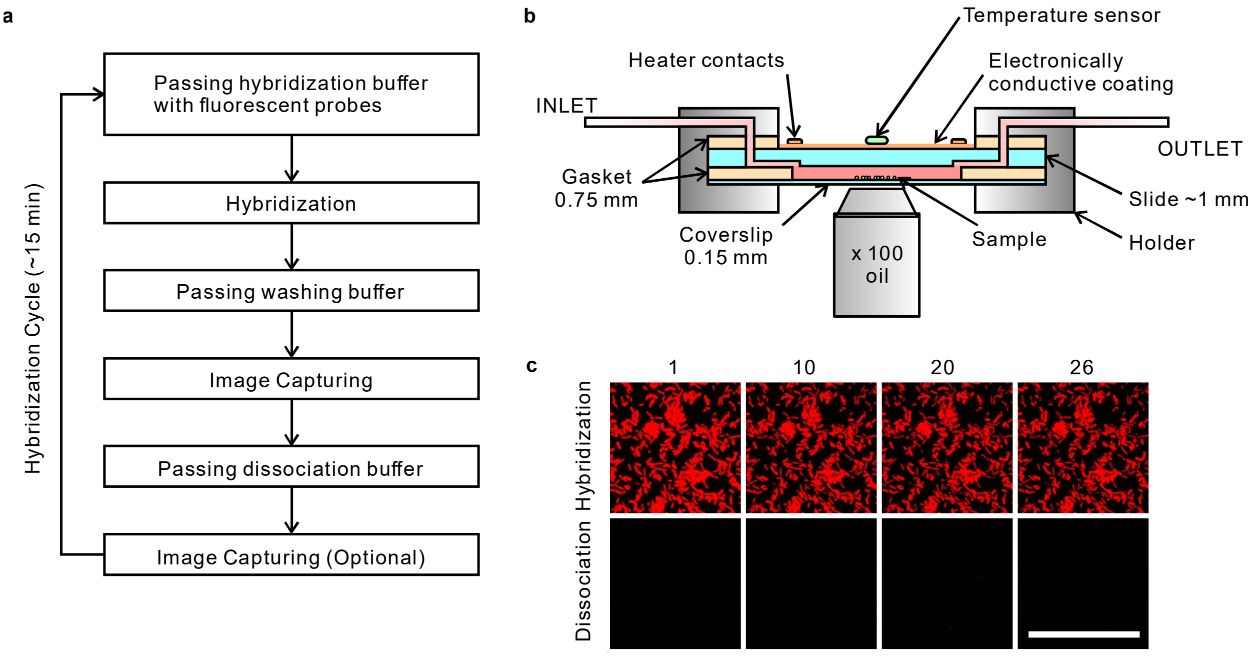

Supplementary Fig. 1. Sequential FISH imaging of microbial communities. a, Workflow of SEER-FISH experiments. For each round of hybridization, hybridization buffer with fluorescent probes flow through the sample for 2 min, flow is stopped and sample is incubated for 3-15 min at 46°C according to the type of sample and the probe concentration used. Then sample is rinsed with washing buffer for 2 min at 46°C to eliminate residual and nonspecific binding of probes. Images are captured right after the washing. After image acquisition, dissociation buffer is flowed through the samples at 46°C for 2 min to strip off hybridized probes. Then dissociation image is captured with the same parameter above for checking the dissociation efficiency. The whole hybridization cycle can be finished in ~15 to 30 min and repeated for N rounds. **b,** Schematic diagram of SEER-FISH experimental setup. A flow chamber (Bioptech FCS2) is secured into a stage adapter to interface with a microscope for imaging. Silicone gasket (40 mm round, 0.75 mm thick) with a rectangle cavity internal that separates the micro-aqueduct slide from the coverslip (40 mm round, 0.15 mm thick) is used to create an optical cavity in the chamber. Laminar flow perfusion that comes into one of the ports (INLET) on one side of the chamber is collected within the optical cavity and then directed out of the chamber on the other side (OUTLET). Uniform temperature across the entire field is maintained by a temperature controller. Flow through this chamber is controlled via an extraneous peristaltic pump. **c,** Representative images of microbes after multiple rounds of hybridization and dissociation (hybridization round 1, 10, 20, and 26). Dissociation images demonstrate the efficient removal of fluorescent probes. Scale bar, 50 μm.

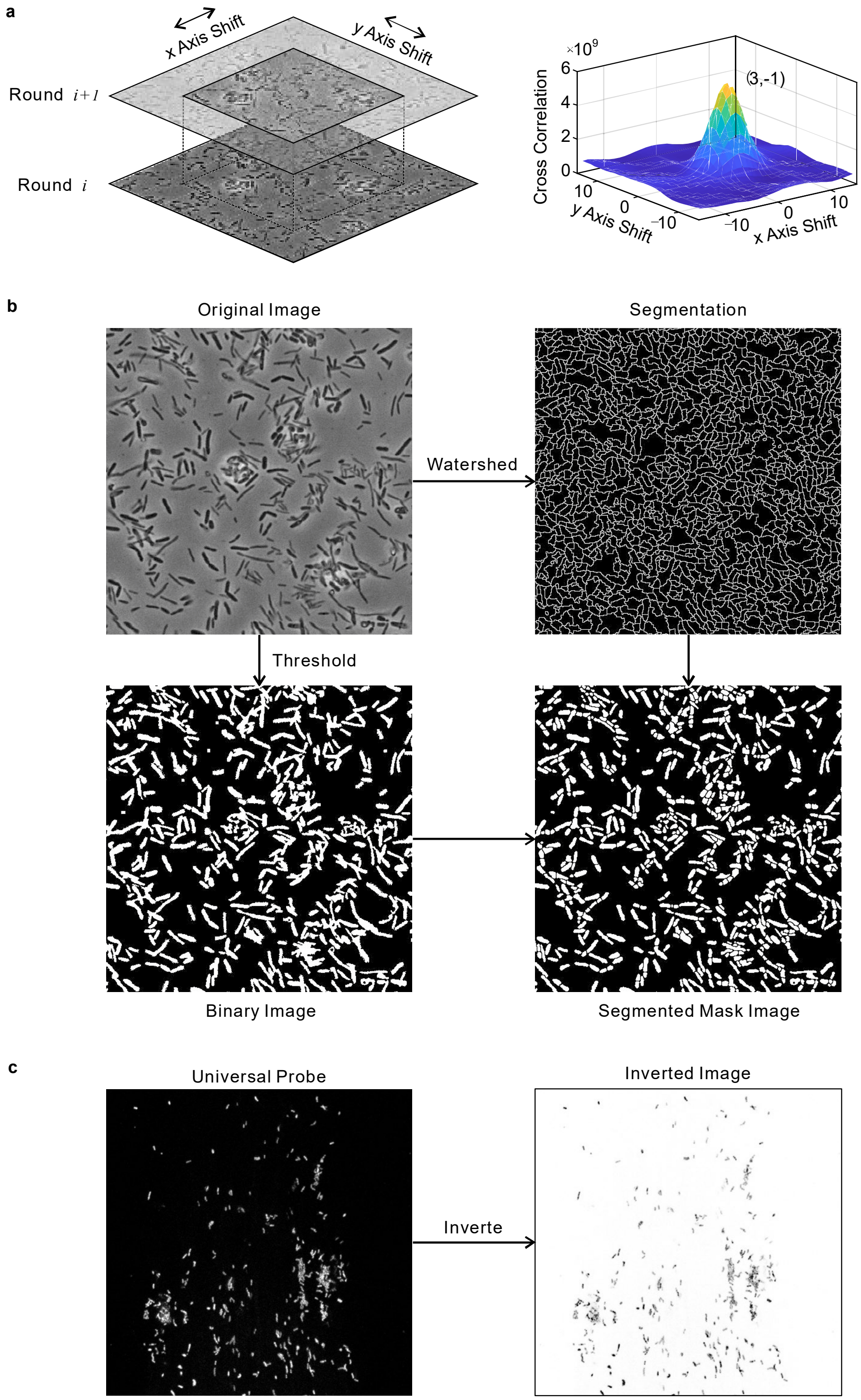

Supplementary Fig. 2. Workflow for image alignment and segmentation of bacterial cells. a, The phase contrast images from neighboring rounds are used for alignment. Multiple images were cropped from the center of the round i+1 image with various shift pixels on x and y axis (left panel). The cross correlation between each cropped image and the image of round i is calculated to find

cropped image with maximal cross correlation (right panel). The images were aligned by shift the image of round i+1 with the same distance as the cropped image. **b,** Identification of single bacterial cells by segmentation. For *in vitro* microbiome samples, the binary image is generated with the phase contrast image of the first-round imaging by adaptive threshold. The segmentation is generated by applying the watershed algorithm on the phase contrast image. The final segmented mask image is generated by applying segmentation on the binary image. Images are analyzed by custom MATLAB scripts. **c,** For *in vivo* microbiome samples, a fluorescent image is acquired by labeling bacteria with the universal probe EUB338, and the inverted image is used for segmentation as shown in panel b.

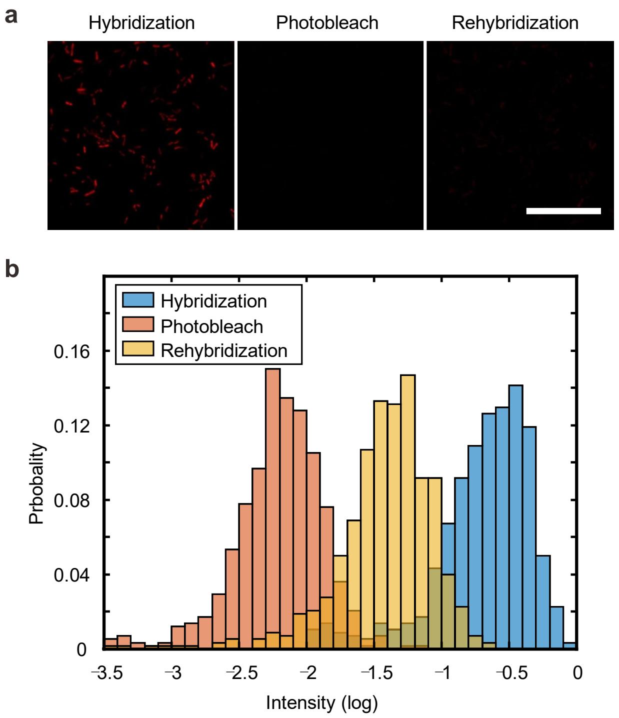

Supplementary Fig. 3. The rehybridization efficiency is low after photobleaching. The mixture of 12 bacterial species was labeled by the universal probe (EUB338-Cy5). Fluorescence was eliminated by photobleaching with a high power laser for 2 min. After photobleaching, the sample was rehybridized with the same probe. **a,** The fluorescent images after hybridization, photobleaching, and rehybridization. Scale bar, 25 µm. **b,** The distribution of fluorescent intensity of bacterial cells after hybridization, photobleaching, and rehybridization. The mean fluorescent intensity decreased about 30 folds after photobleaching and only recovered to 20% of the original level after rehybridization. Source data are provided as a Source Data file.

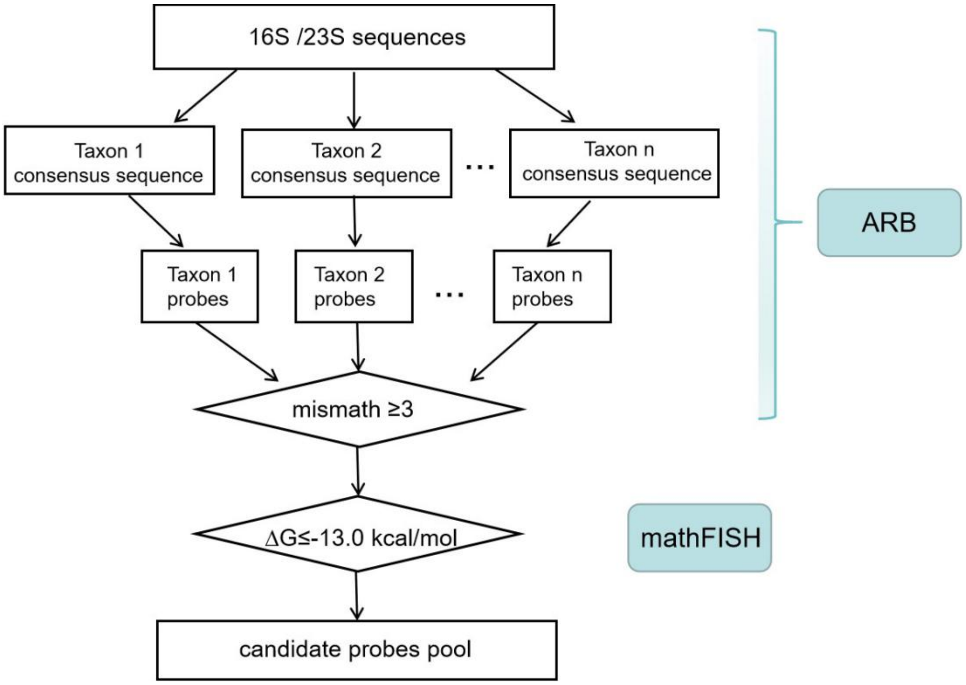

Supplementary Fig. 4. Design of taxon-specific probes that target bacterial rRNA. The 16S or 23S rDNA sequences of targeted bacterial taxa are built into a local database and imported to the ARB program for probe design. Probes with at least three central mismatches to all non-target taxa are retained. Furthermore, ΔG for probes binding to targets is calculated by mathFISH. Probes with a ΔG value less than -13.0 kcal/mol are chosen as candidate probes.

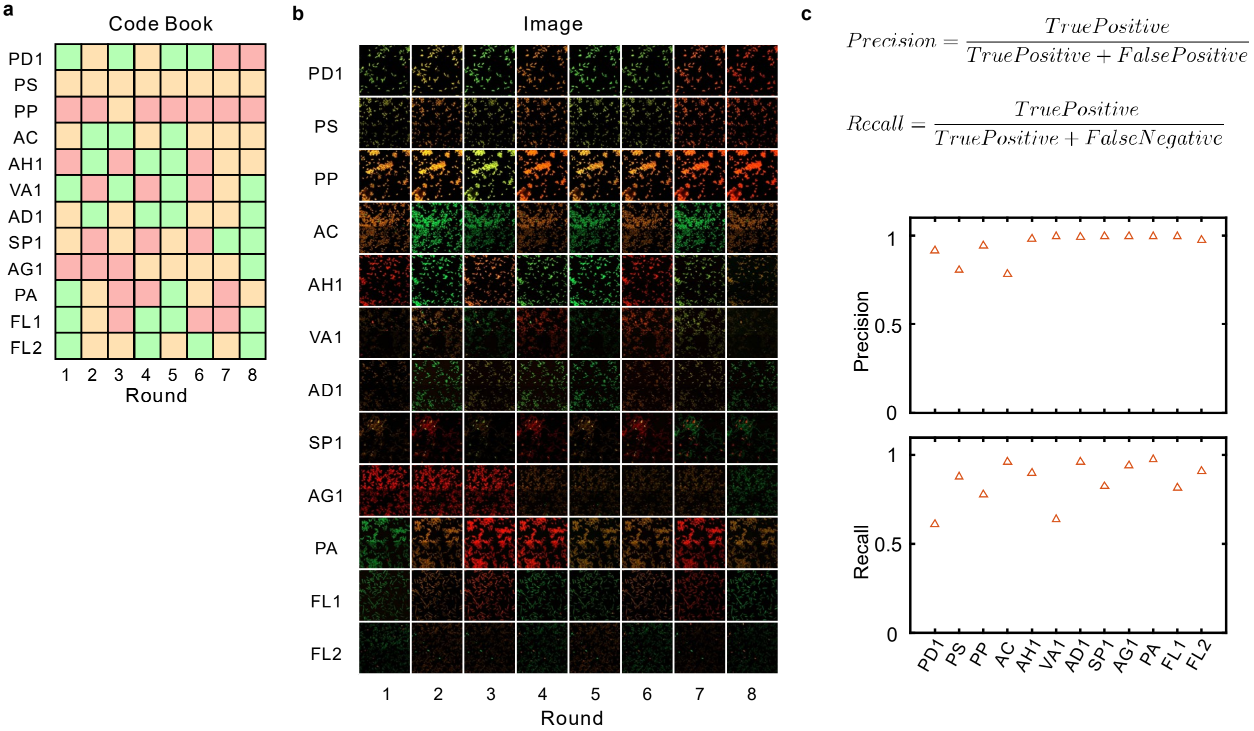

Supplementary Fig. 5. Accuracy evaluation of SEER-FISH. a, The codebook used for the experiment in Fig. 2c (R=8, HD≥4, S=12, F=3, Supplementary Table 5). The same codebook is also shown in Fig. 2a. **b,** The fluorescent images of each species during eight hybridization rounds. Fixed bacterial suspensions of 12 species were separately spotted onto 40-mm, #1.5 coverslip and air-dried. 8-round SEER-FISH was implemented according to the codebook in panel a. The fluorescence and phase contrast images of each species were acquired after each round of hybridization. c, Precision and recall of each bacterial species, based on identification of the 8-bit barcodes (see Precision and recall calculation in Methods). Source data are provided as a Source Data file.

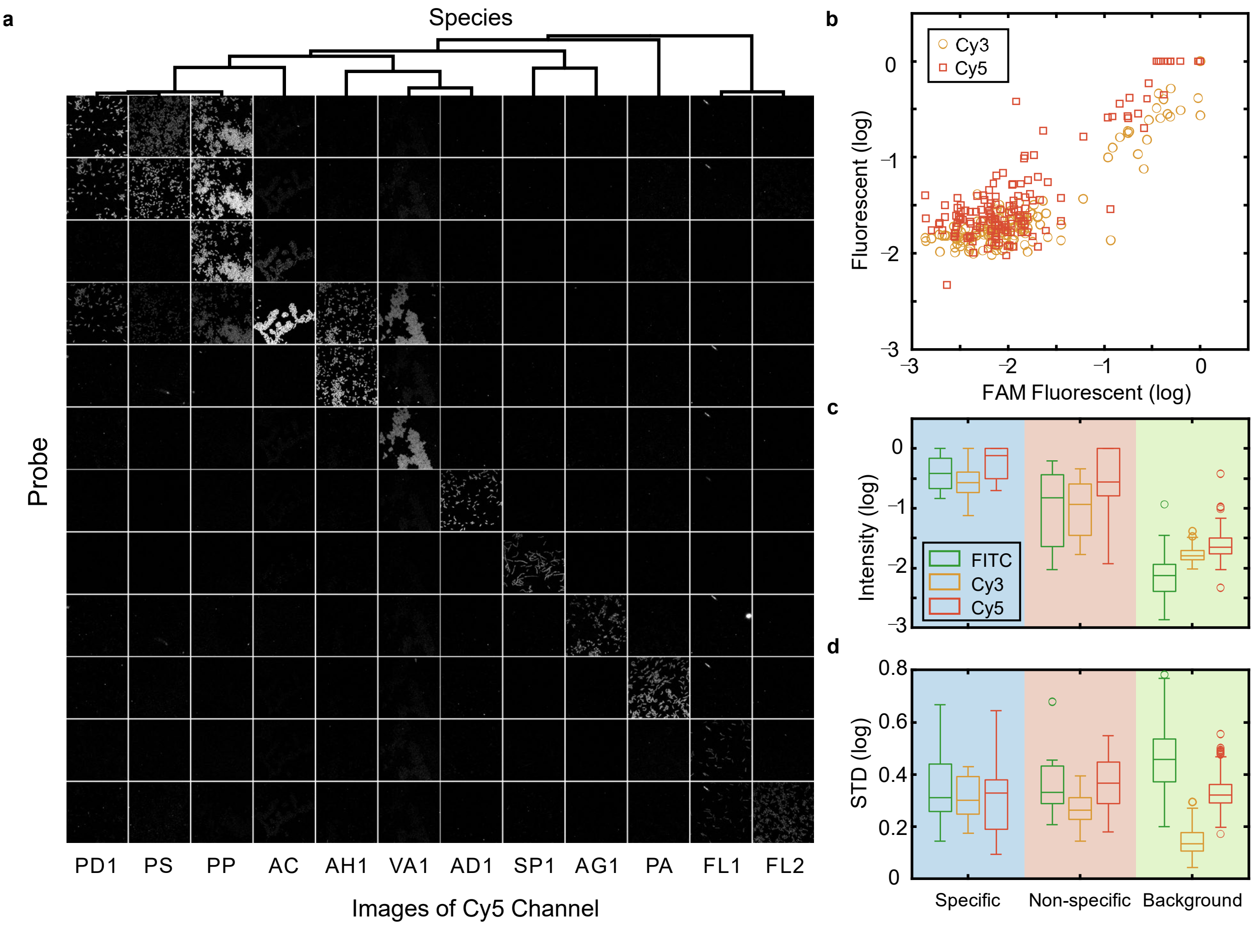

Supplementary Fig. 6. Probe specificity analysis. a, Fluorescent images of 12 pure bacterial cultures when hybridized with 12 candidate probes. 12 candidate probes were chosen to hybridize with pure cultures of target and non-target bacteria according to the codebook in Supplementary Table 5 ("codebook used for probe specificity analysis"). Each row represents the specific probe designed for each strain, ranked in the same order as columns. To check the consistency of fluorescence signals between different fluorophores, 12 specific probes with three different conjugations (FAM, Cy3, Cy5) were all examined during 12 rounds of FISH imaging (see Supplementary Table 5). The images of three fluorescence channels and phase-contrast images were collected for all species. The images shown were based on probes labeled with Cy5 fluorophore. The fluorescence intensity of displayed images was normalized by the highest intensity in each column. **b,** The fluorescent intensity of probes conjugated to Cy3 or Cy5 is proportional to the intensity of probes conjugated to FAM. Pearson correlation is 0.88 (Cy3 to FAM, orange circles) and 0.90 (Cy5 to FAM, red squares), respectively. **c,** The mean fluorescent intensity in different channels. The probe-species pairs are grouped into 3 groups based on ΔG calculated by mathFISH. Three colored regions indicate specific binding (ΔG < -13.0 kcal/mol, n=12), non-specific binding (-7.3 kcal/mol > ΔG >-13.0 kcal/mol, n=14) and background (ΔG >-7.3 kcal/mol, n=118), respectively. The box plot indicates the interquartile range (25% to 75%) in each group. **d,** The standard deviation of fluorescent intensity in different channels. For panel c and d, each box and the line inside the box labeled the first quartile, third quartile and median of

each set of data. The length of the whiskers is set to 1.5 folds of the height of the box. Source data are provided as a Source Data file.

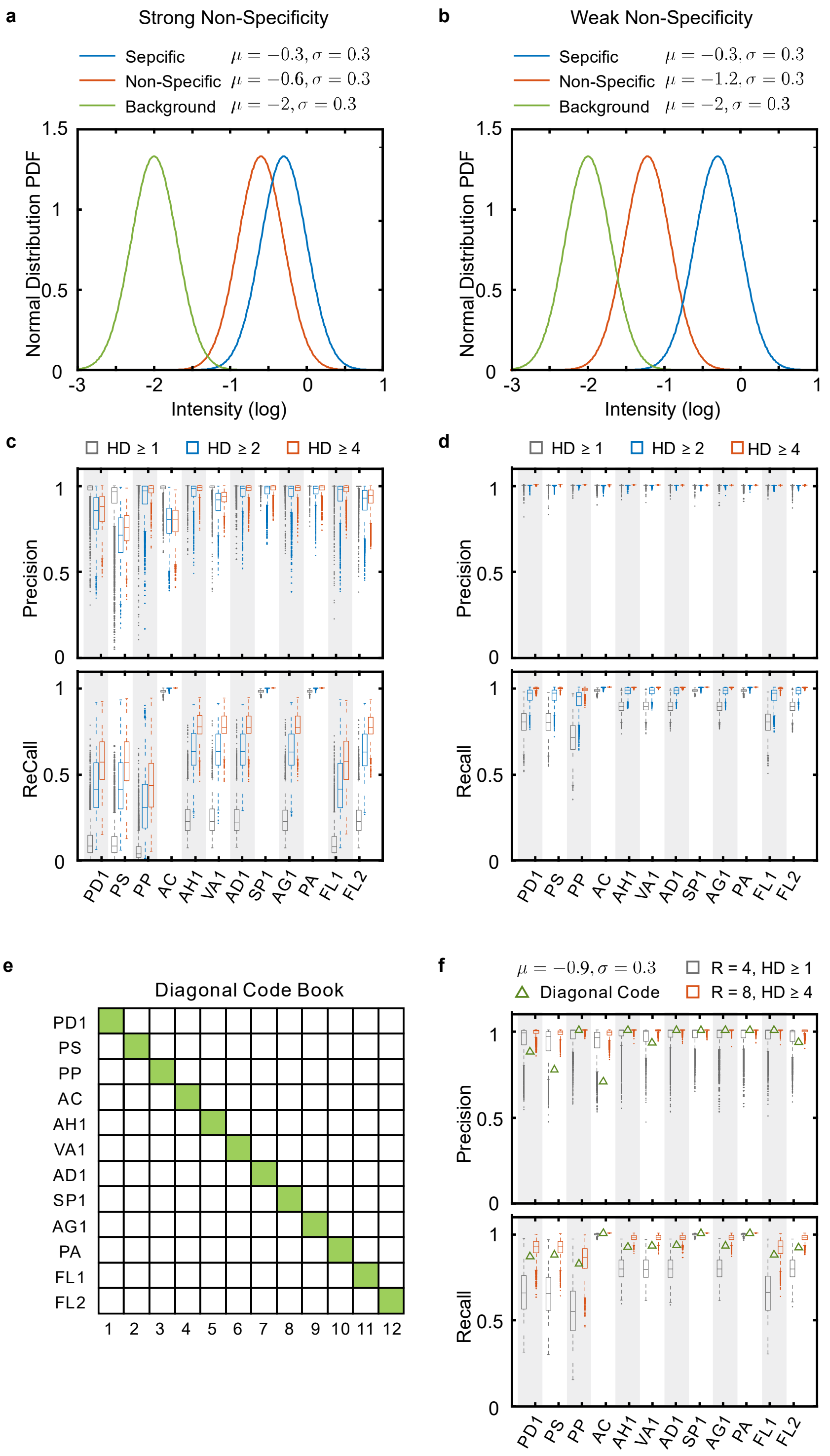

Supplementary Fig. 7. Error-robust encoding enables improved accuracy. a and **b,** The mean intensity (log scale) of probe-species pairs from non-specific group is set as μ = -0.6 (a, strong non-specificity) and μ = -1.2 (b, weak non-specificity) respectively. **c** and **d,** The precision and recall of 12 species obtained by simulations with different codebooks for the parameters used in a and b, respectively. The colored boxplot indicates the predicted distribution of precision and recall of SEER-FISH with n=5000 randomly generated codebooks (F=3, R=8) with various minimal HD.

Each box and the line inside the box labeled the first quartile, third quartile and median of each set of data. The length of the whiskers is set to 1.5 folds of the height of the box. The outliers are plotted as dots. Error-robust encoding schemes (HD≥2 and HD≥4) improve the overall performance of taxonomic identification. **e,** The diagonal codebook is a trivial codebook that uses N rounds of imaging to barcode N taxa. During each round, only one probe is present, and the barcode of each cell can be simply identified by finding the brightest round (without error-correction). **f,** The predicted precision and recall of taxon identification by exemplary coding schemes, including R4HD1, R8HD4 and the diagonal codebook. The boxplot indicates the predicted distribution of precision and recall of SEER-FISH with n=5000 randomly generated codebooks (R4HD1, gray; R8HD4, red). The green triangles indicate the predicted precision and recall of the diagonal codebook illustrated in panel e. Each box and the line inside the box labeled the first quartile, third quartile and median of each set of data. The length of the whiskers is set to 1.5 folds of the height of the box. The outliers are plotted as dots. The mean intensity (log scale) of probe-species pairs for specific, non-specific and background hybridization is set as -0.3, -0.9 and -2, respectively. Source data are provided as a Source Data file.

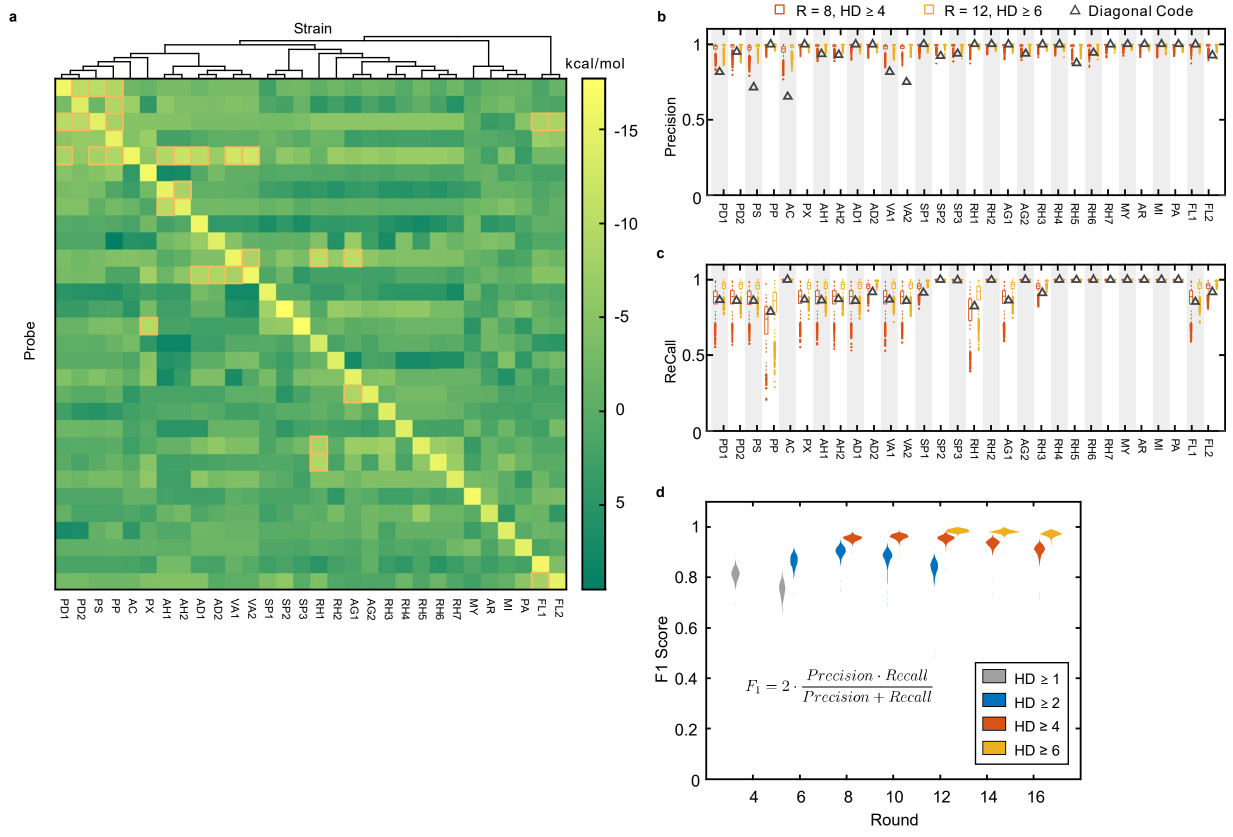

Supplementary Fig. 8. Simulated performance of SEER-FISH for SynCom30. a, The ΔG of each probe-specie pair was calculated by mathFISH. The pairs on the diagonal (ΔG <-13.0 kcal/mol) are grouped as specific binding. The pairs marked by orange boxes (-7.3 kcal/mol > ΔG >-13.0 kcal/mol) are grouped as non-specific binding. The rest (ΔG>-7.3 kcal/mol) are grouped as background. **b** and **c,** The precision and recall of 30 species obtained by simulation with 2 sets of n=5000 randomly generated codebooks (Orange boxplot, F=3, R=8, HD≥4, S=30; Yellow boxplot, F=3, R=12, HD≥6, S=30) and the diagonal codebook (gray triangles). The diagonal code book is similar to the code book shown in Supplementary Fig. 7e (30 imaging rounds for 30 strains). Each box and the line inside the box labeled the first quartile, third quartile and median of each set of data. The length of the whiskers is set to 1.5 folds of the height of the box. The outliers are plotted as dots. The mean intensity (log scale) of probe-species pairs for specific, non-specific and background hybridization is set as -0.3, -0.9 and -2, respectively. **d,** Simulated performance of codebooks with varying levels of hybridization round (R, x-axis) and Hamming distance (HD, specified by colors). F1 score for 5000 randomly drawn codebooks (F=3, S=30) is shown. As expected, for a given HD, F1 score decreases with R; for a given R, F1 score increases with HD. The log-transformed fluorescent intensity (log(Ppi)) is drawn from a normal distribution with the mean fluorescence intensity of 10-0.3, 10-0.9 and 10-2 for specific binding (ΔG < -13.0 kcal/mol), non-specific binding (-7.3 kcal/mol > ΔG >-13.0 kcal/mol) and background (ΔG >-7.3 kcal/mol), respectively. Source data are provided as a Source Data file.

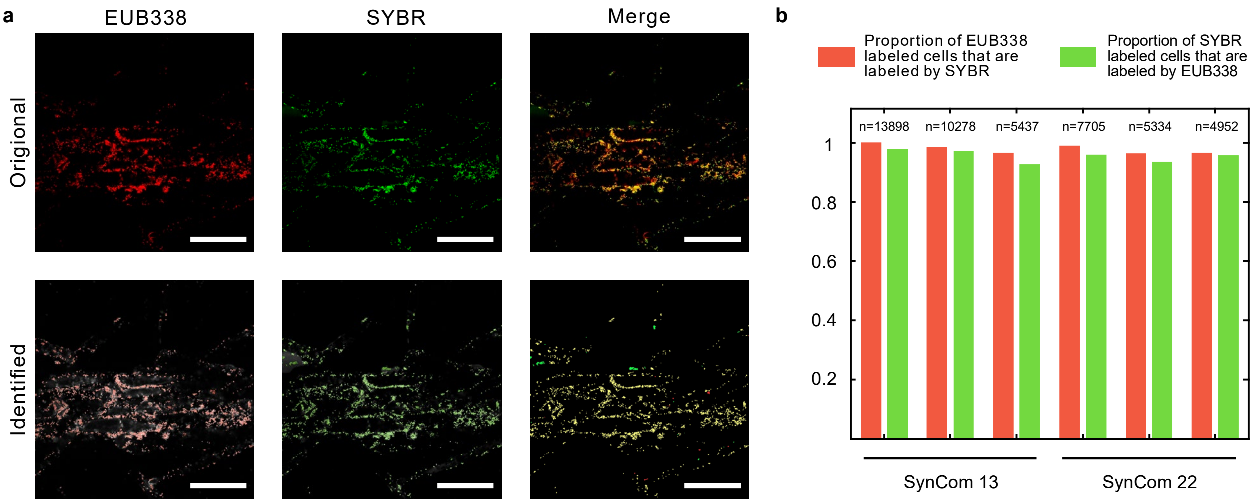

Supplementary Fig. 9. Identification of root-colonized bacteria. Root-colonized bacterial cells recognized by universal FISH probe EUB338 were validated by staining with SYBR Safe (Invitrogen). Roots colonized with a 13-species community (SynCom 13) or a 22-species community (SynCom 22) were hybridized with EUB338 probe for 10 min (20% formamide, 46℃), then stained with SYBR Safe (10X) for 15 min at room temperature before imaging. **a,** Representative images of bacteria-colonized root samples labeled by the FISH probe EUB338 and the nucleic acid dye SYBR Safe. Single cells labelled by EUB338 or SYBR Safe are shown below the original images. **b,** Proportion of EUB338 labeled cells that are labeled by SYBR Safe (red bars) and vice versa (green bars) across multiple roots. The total number of cells imaged for each root is indicated. 97.8%±1.5% EUB338 labeled cells were labeled by SYBR Safe; 95.5%±2.0% SYBR Safe labeled cells were labeled by EUB338. Scale bars, 50 μm. Source data are provided as a Source Data file.

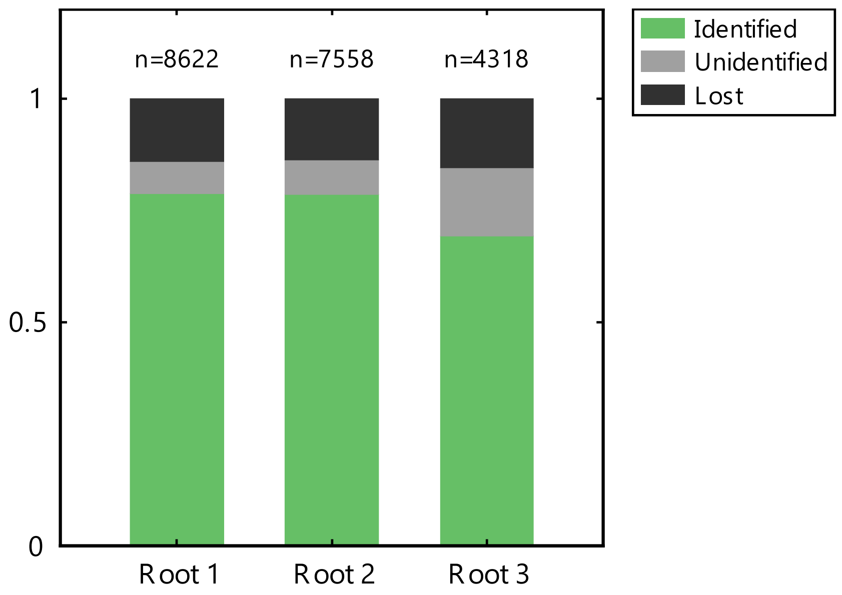

Supplementary Fig. 10. The fraction of lost and unidentified bacterial cells in multi-round FISH imaging of *Arabidopsis* roots. The fraction of identified, unidentified and lost bacterial cells on 3 roots. The number of bacterial cells detected by the universal probe EUB338 is indicated on top. A bacterial cell is labeled as "lost" if it is not detected in any fluorescence channel for more than 3 rounds. Source data are provided as a Source Data file.

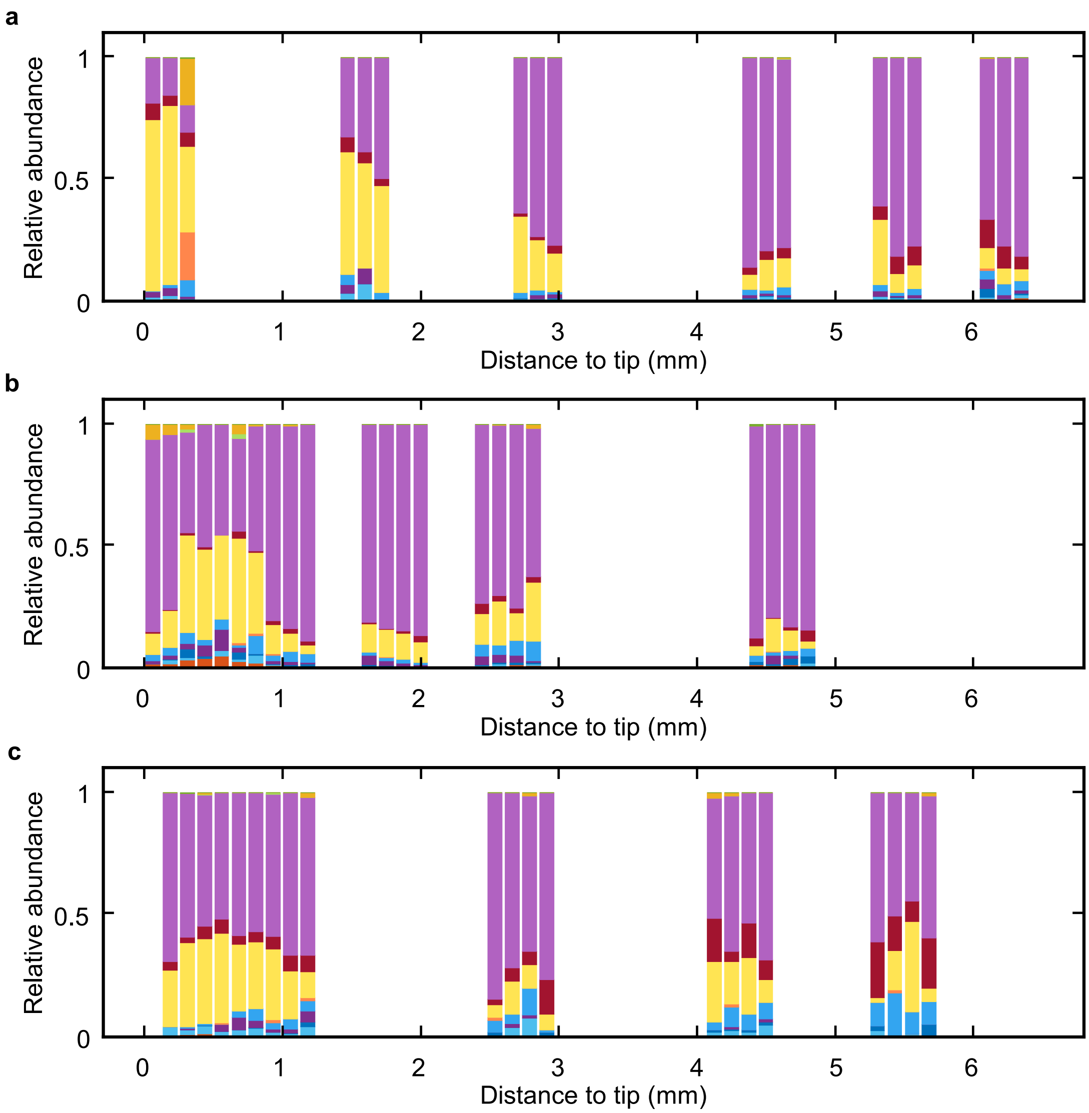

Supplementary Fig. 11. Colonization of microbial communities on *Arabidopsis* roots. The composition of microbial communities colonized on multiple regions of 3 roots (**a**, Root 1; **b**, Root 2; **c**, Root 3) was measured by SEER-FISH. Images are shown in Fig. 4. Analysis of Root 3 is shown in Fig. 5a-d. Each bar represents the community composition of the FOV with 125 µm (height）× 250 µm (width). Source data are provided as a Source Data file.

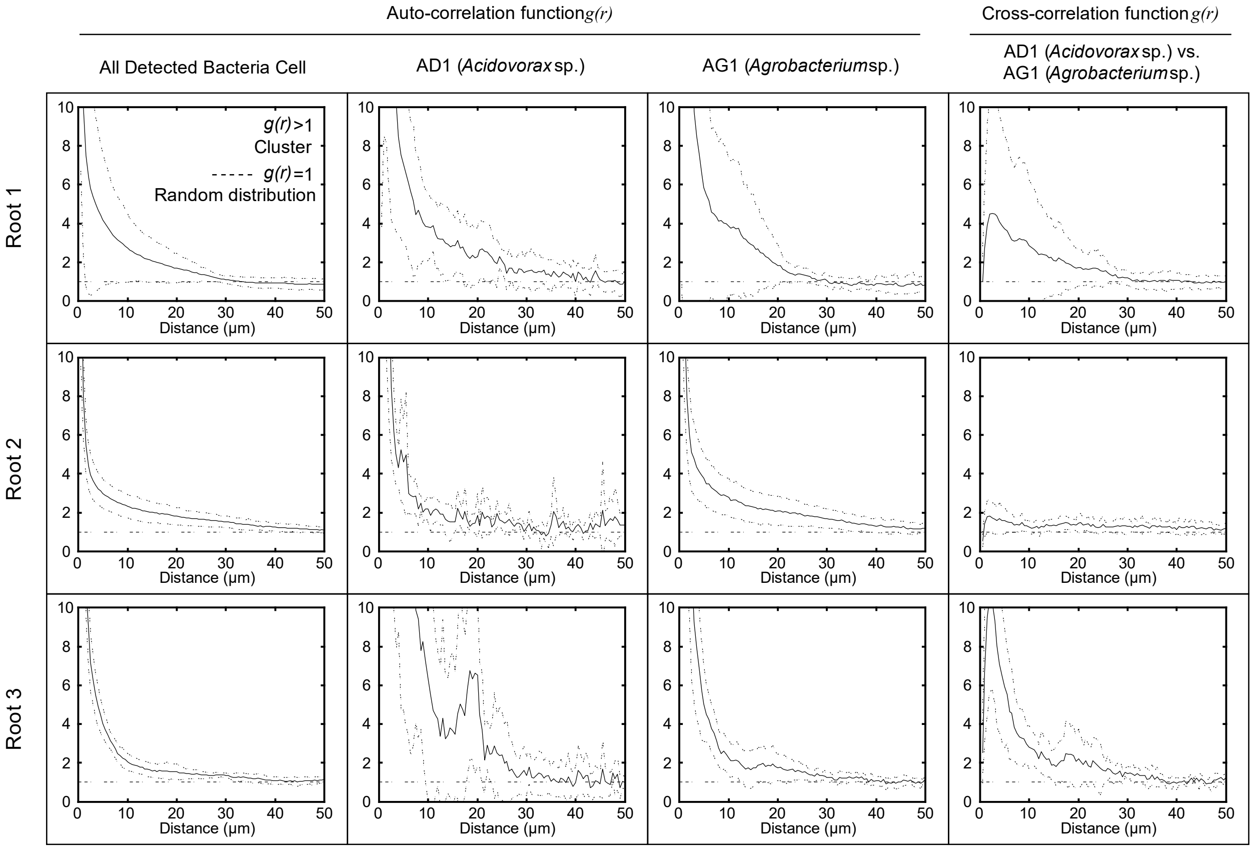

Supplementary Fig. 12. Clustering analysis of microbial communities on *Arabidopsis* roots. The aggregation of bacterial cells is analyzed by the linear dipole algorithm (see Clustering analysis in Methods). The solid lines indicate the pair auto-correlation or cross-correlation between bacterial cells, the shadows indicate the 95% confidence intervals estimated by sampling different regions on each root. The horizontal dash line (g(r)=1) refers to the expected value of a randomized spatial distribution. Source data are provided as a Source Data file.

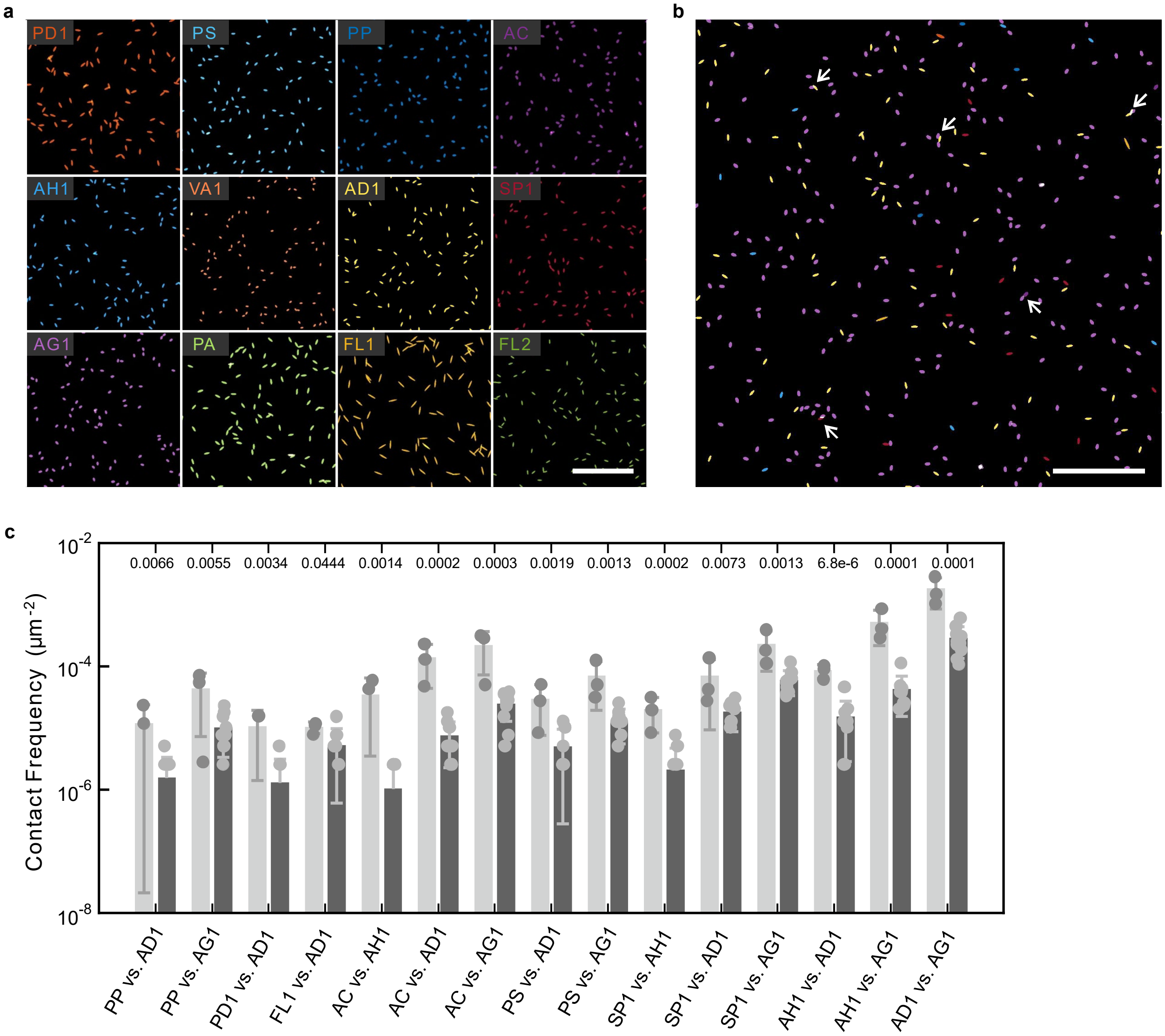

Supplementary Fig. 13. Model cells to determine the frequency of random associations. a, Representative images of simulated model cells. Bacterial cells were modeled as ellipses; the lengths of major and minor axes were determined by images (Fig. 2c). Scale bar, 25 μm. **b,** Simulation of contact frequency of randomly distributed cells. Contacts in the representative simulated image were indicated by white arrows. The density of each species was determined by the measured density on root samples. Scale bar, 25 μm. **c,** The mean contact frequency between different species in simulations (dark gray) and on roots (light gray). Statistically significant associations (unpaired one-sided *t*-test, p-values are listed on top of the bar plots) were shown. Error bars indicate the standard deviation of replicates (n=3, zero data not shown) or independent simulations (n=10). Source data are provided as a Source Data file.

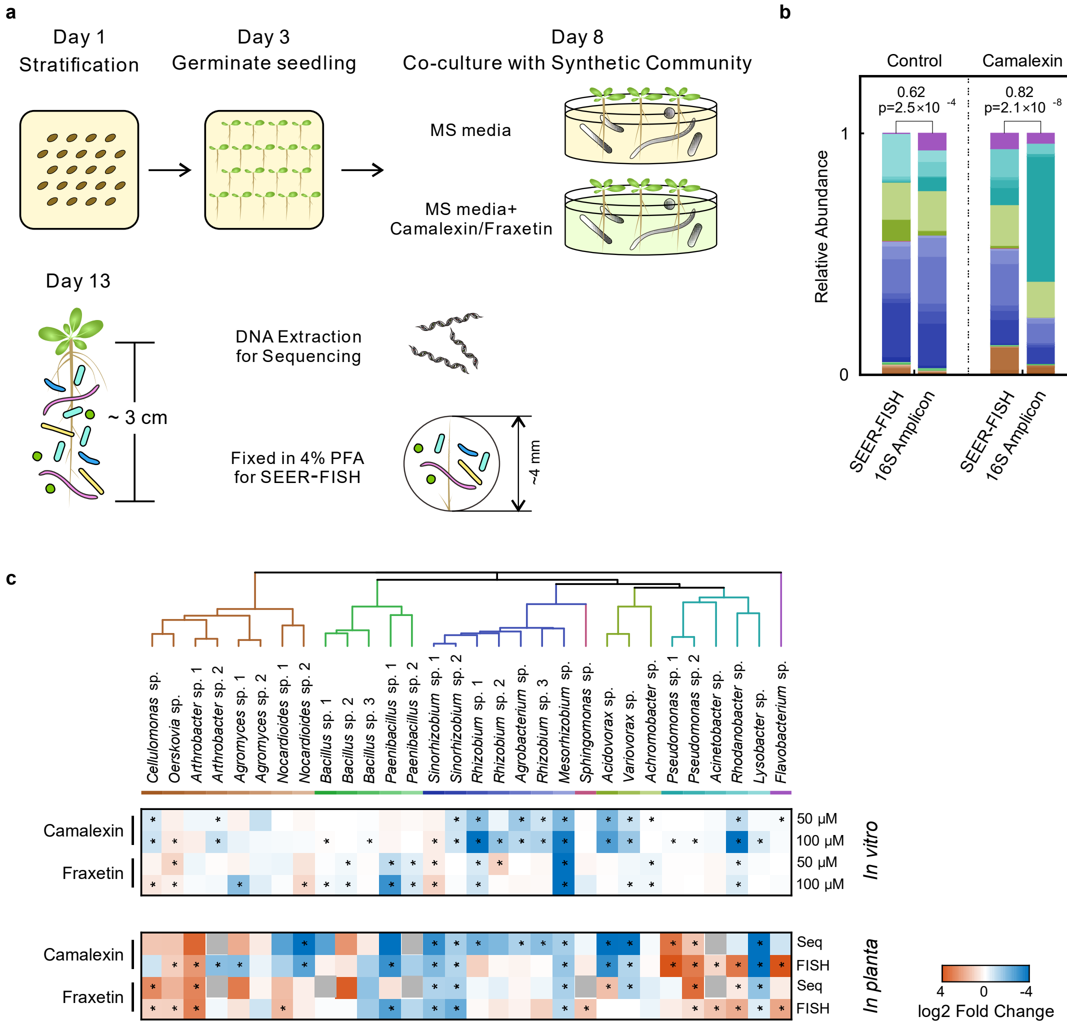

Supplementary Fig. 14. Perturbation on the community profile by camalexin and fraxetin. **a,** Illustration of the experiment setup. A synthetic community of 30 strains (SynCom30.2) was used to colonize Arabidopsis. For treatment groups, camalexin or fraxetin was introduced into MS media. **b,** The composition of the 30-strain synthetic microbial community colonized on root measured by SEER-FISH (n=10, within ~4mm from root tip) and by 16S amplicon sequencing (n=3 samples, in each sequencing sample 4 roots were pooled for DNA extraction). The Spearman correlation between the compositional profile given by SEER-FISH and the profile given by 16S amplicon sequencing (***: p<0.001) is shown for each experimental group. **c,** Fold change in the abundance of 30 bacterial strains under treatment of plant metabolites in vitro (monoculture, measured by Optical Density) and in planta. Seq and FISH indicate the fold change in abundance measured by sequencing and by imaging. Gray areas: fold change is not applicable due to low abundance. *: P<0.05, unpaired two-side Student's t-test. Source data are provided as a Source Data file.

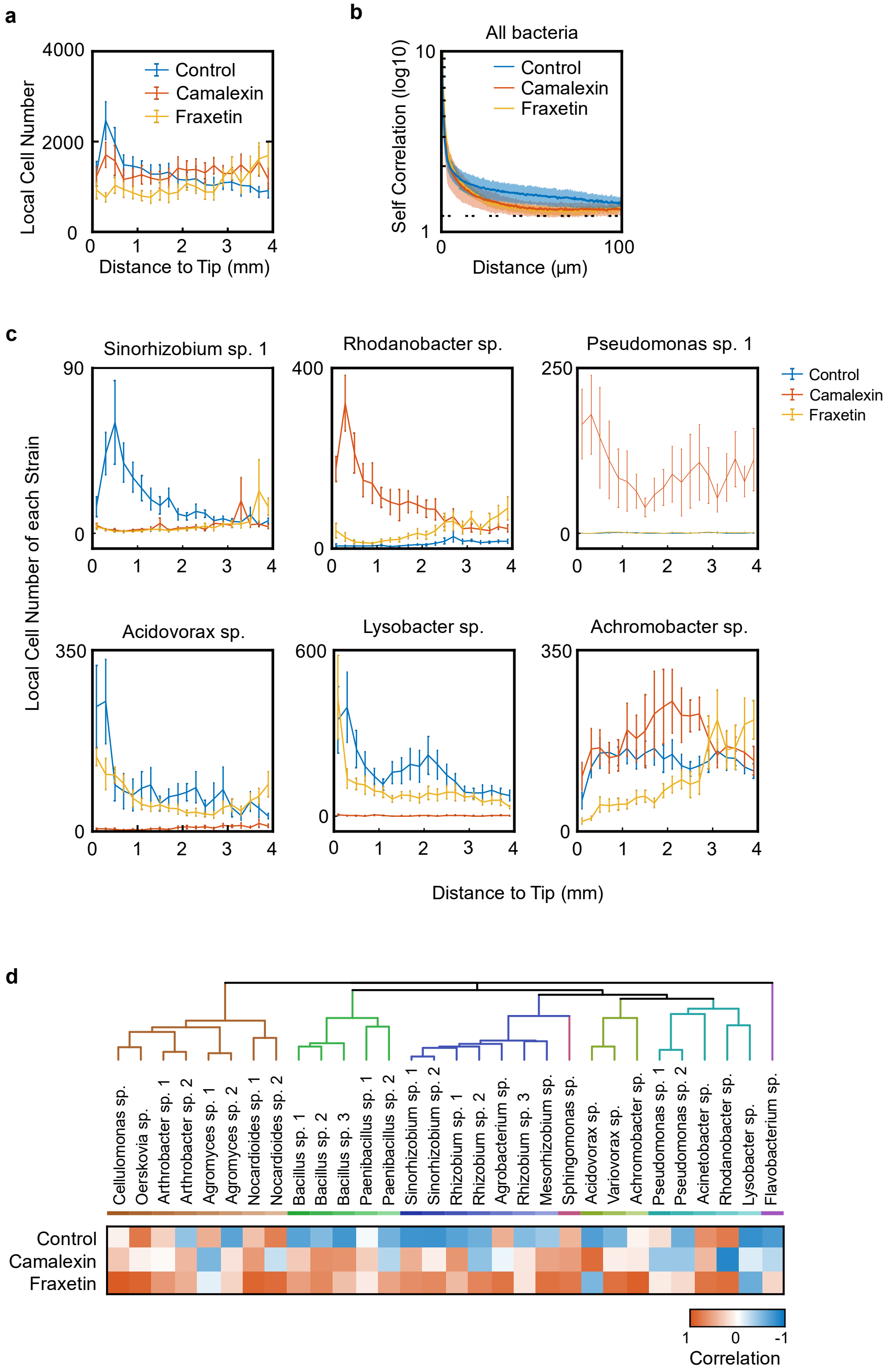

Supplementary Fig. 15. Spatial distribution of microbial taxa along roots. a, The spatial distribution of microbial cells identified by SEER-FISH. Data are presented as mean values +/- SEM by sampling n = 10 roots. **b,** The spatial self-correlation of root-colonized bacterial cells is

analyzed by linear dipole algorithm (see Clustering analysis in Methods). The solid lines indicate the mean correlation between bacterial cells, the shadows indicate the 95% confidence intervals (estimated by sampling n=10 roots). The horizontal dashed line (g(r)=1) refers to the expected value of random distribution. **c,** Spatial distribution of 6 representative strains. Data are presented as mean values +/- SEM by sampling n = 10 roots. **d,** For each experimental group, Pearson correlation is calculated between the local cell number (each local area is 200 um in length) and the distance to root tip. Positive (or negative) correlation means that the local abundance of the corresponding strain increases (or decreases) with the distance to root tip. Source data are provided as a Source Data file.

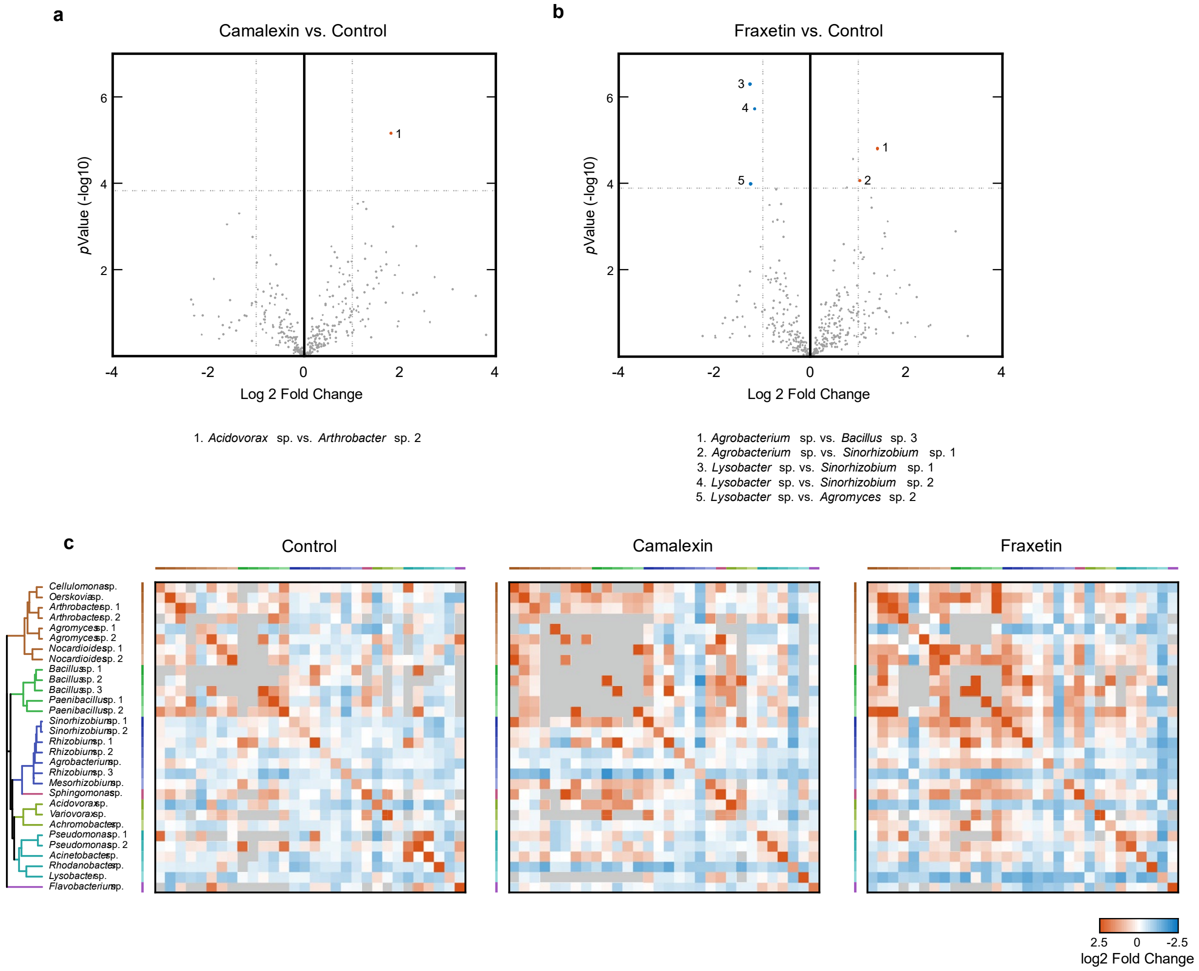

Supplementary Fig. 16. Spatial association analysis of root-colonized microbial communities. a and **b,** Differential spatial association between camalexin-treated and control group (panel a), or between fraxetin-treated treated and control group (panel b). The horizontal dashed line indicates the significance threshold determined by Bonferroni correction (see Spatial association analysis in Methods). Statistically significant spatial associations (unpaired two-side *t*-test with Bonferroni correction) are listed below each panel. **c,** Spatial association network of each experimental group (see Spatial association analysis in Methods). Source data are provided as a Source Data file.

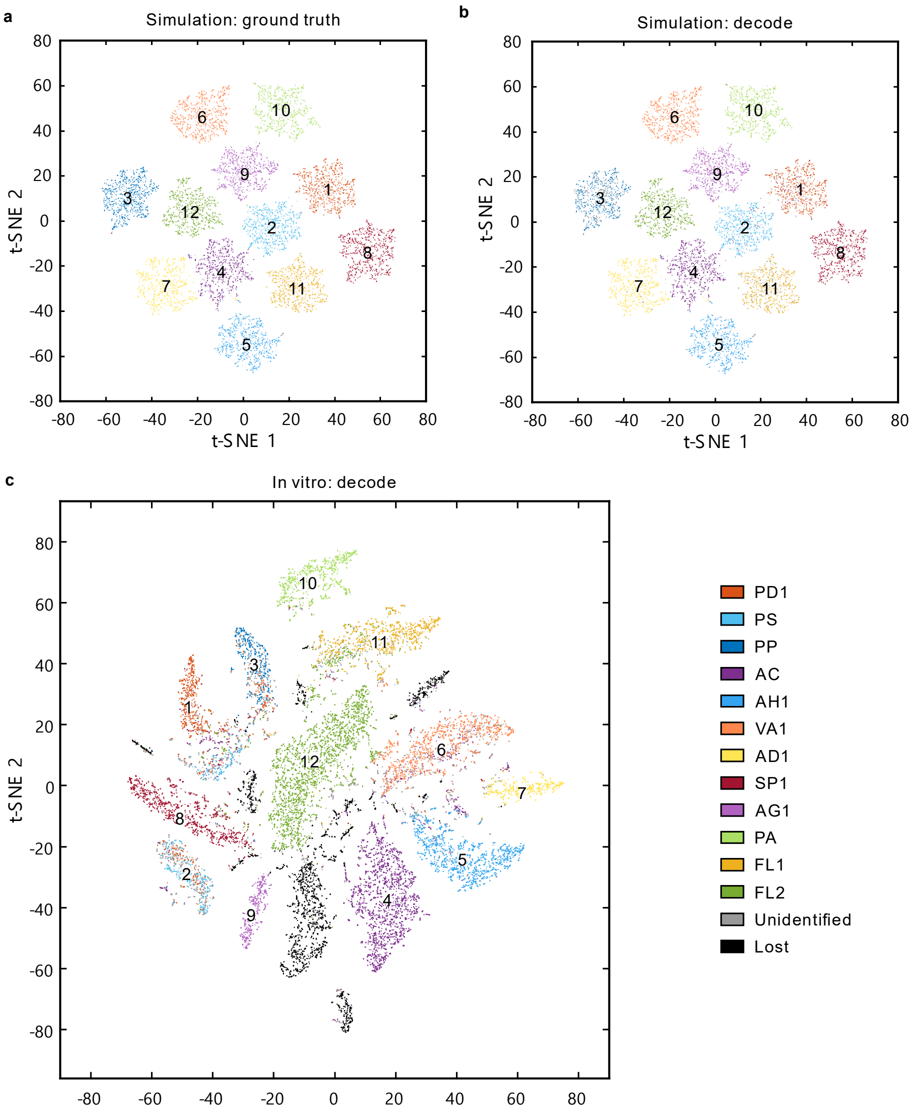

Supplementary Fig. 17. Dimensionality reduction analysis of the multi-round, multi-color imaging data acquired by SEER-FISH. a and **b,** t-SNE analysis of the simulated data of a 12-species synthetic community. The 8-round 3-color codebook is the same as Supplementary Table 5. The specificity parameters for specific, non-specific and background hybridization is set as -0.3, -0.9 and -2 according to the ΔG, respectively. For each data point (i.e. bacterial cell), the 24 dimensional data (8 imaging rounds × 3 fluorescence channels) is reduced to 2 dimensions by 'tsne' function in MATLAB (parameters are set as, LearnRate: 200; Perplexity: 20; Exaggeration: 10). The points are colored by species known as ground truth (in panel a) or inferred by our encoding-decoding strategy (in panel b). Unidentified cells are labeled as grey dots in panel b. **c,** t-SNE analysis of the imaging data of 12 species (data from Fig. 3a). The points are colored by identified species. Unidentified and lost cells are labeled as grey and black dots, respectively. Source data are provided as a Source Data file.

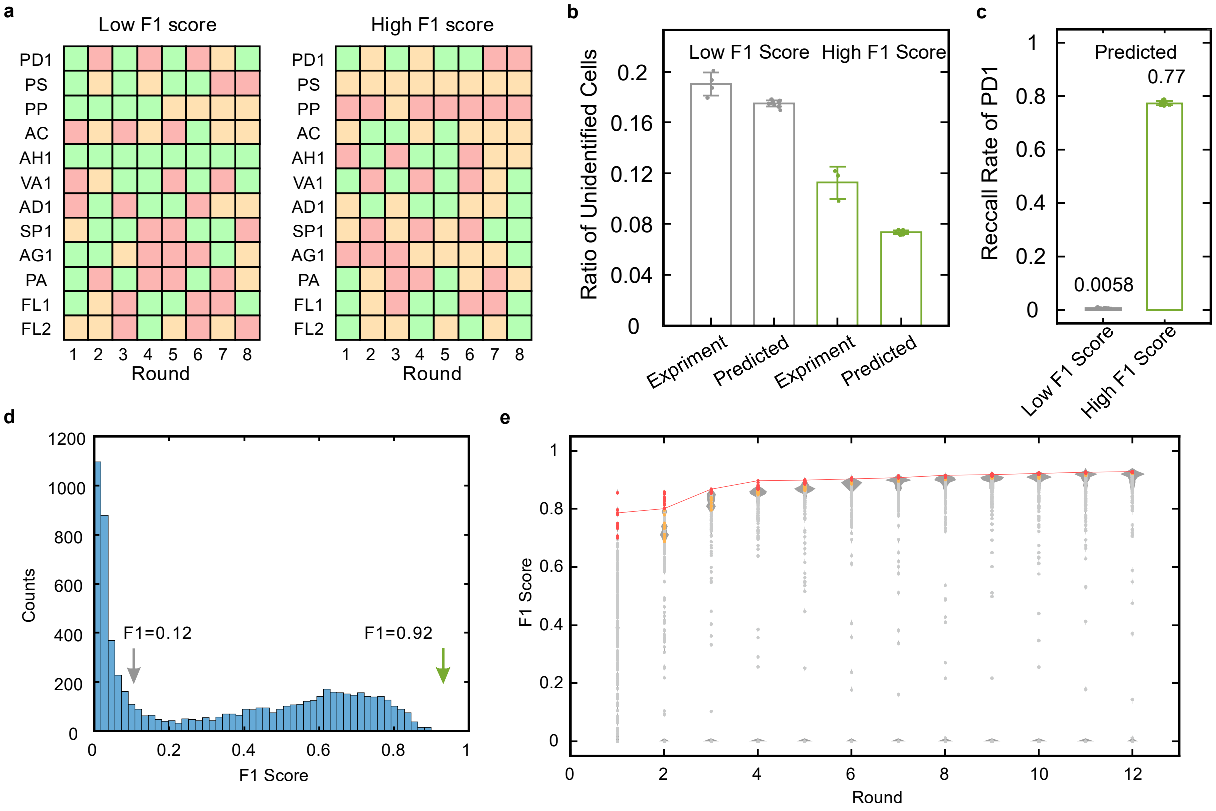

Supplementary Fig. 18. Codebook optimization. a, Two codebooks with low and high predicted F1 score. **b,** The mean fraction of unidentified cells in experiments and simulations, for 2 set of codebooks. Error-bars are the standard deviation of FOVs (n⩾3) in experiments or independent simulations (n=10). **c,** The mean recall rate (n=10) of PD1 for 2 set of codebooks. Error-bars are the standard deviation of n=10 independent simulations. **d,** The distribution of predicted F1 score of 10000 randomly generated R8HD4 (S=12) codebooks. To simulate the F1 score, the fluorescence intensity P_{pi} (probe p hybridized to species i) is set by experimental measurement (Supplementary Fig. 6, see Simulations of SEER-FISH in Methods). The codebook used in Fig. 2 has a high predicted F1 score (green arrow). **e,** The optimization of F1 score via genetic algorithm. The gray dots indicate the F1 score of random generated codebooks at Round 1. The red dots indicate the 15 codebooks with the highest F1 score at each round, and the red line indicates the optimization of the best codebook. The orange dots indicate the 15 best codebooks from the previous round. The green dots indicate the codebooks evolved from the previous round. Source data are provided as a Source Data file.

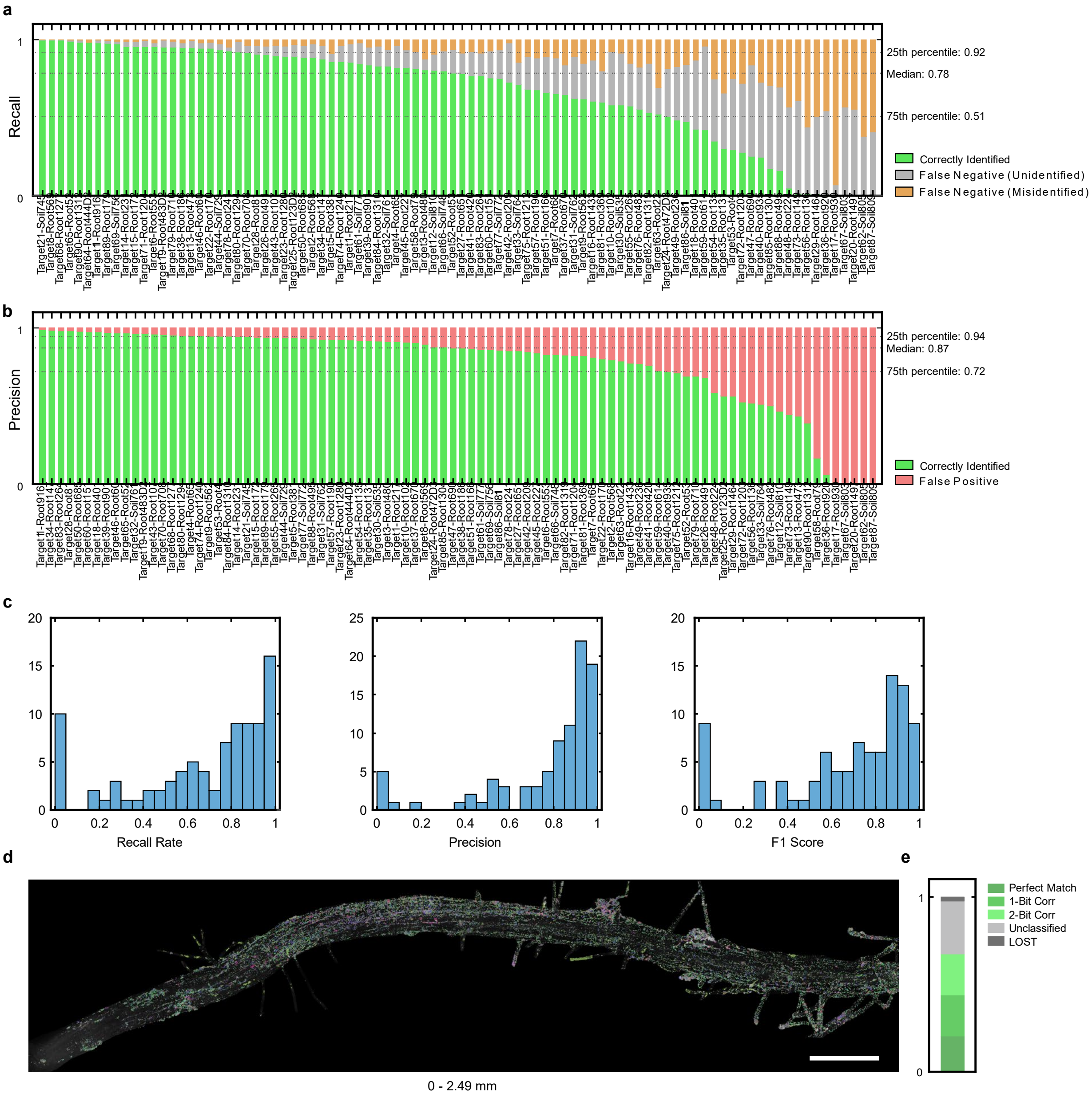

Supplementary Fig. 19. Spatial profiling of a 130-strain microbial community colonized on Arabidopsis root. a, Recall ratio of 90 taxa measured by SEER-FISH with the 9-bit barcodes. For each taxon, cells correctly identified are true positives (Green); cells incorrectly identified as the other 89 taxon are marked as misidentified (Orange); cells that cannot be classified to any of the 90 taxa are marked as unidentified (Gray). **b,** Precision of 90 taxa measured by SEER-FISH with the 9-bit barcodes. Cells of the other 89 taxa incorrectly identified as the corresponding taxon are false positives (Red). Ratios are normalized by the identified cell number of each taxon. The ranking is sorted by recall (panel a) or precision (panel b), respectively. **c,** The 130 strains were grouped into 90 taxa based on the similarity of 16S rRNA sequences. Information of strains and probe sequences is provided in Supplementary Table 3. We used pure cultures of 90 taxa to evaluate the performance of taxonomic identification by SEER-FISH. Each strain was separately

coated onto a coverslip, then hybridized with probes according to the codebook and imaged for nine sequential rounds. The sample pre-treatment, probe concentration, hybridization time, and R9HD4 codebook in 90-taxon validation were the same as used in 130-strain in vivo imaging. Bacterial cells were identified by decoding their barcodes and compared with ground truth. The median and interquartile range of recall rate, precision, and F1 score are 0.78(0.51-0.92), 0.87(0.72-0.94,) and 0.77(0.59-0.90), respectively. **d,** Imaging of a 130-strain synthetic community colonizing on Arabidopsis root. Around 40,000 bacterial cells were identified in 82 FOVs along the root. Numbers below the image indicate the distance to the root tip. Scale bar, 200 µm. **e,** The ratio of correctly identified cells on root was ~65%. Source data are provided as a Source Data file.

Supplementary Table 1. Comparison of FISH-based methods for spatial mapping of microbial communities.

	SEER-FISH	HiPR-FISH	CLASI-FISH
**Multiplexity**	F^N ($F \geq 3$, $N \geq 20$)	2^F-1 ($F \leq 16$)	2^F-1 ($F \leq 16$)
**Error correction**	Yes	No	No
**Speed**	15-30 min per round	8-24 hours	6-20 hours
**Probe costs**	Increase linearly with the number of target taxa and imaging rounds	Economic: 10 types of fluorescent readout probes	Two types of fluorescent probes for each target taxa
**Equipment needs**	Fluidics control for multi-round labeling	Spectral detector	Spectral detector

Supplementary Table 2. Information of bacterial strains used in SynCom12 and SynCom30.

Abb.	Strain ID	Phylum	Class	Family	Genus (species)	Gram staining
***PD1**	root401	*Proteobacteria*	*Gammaproteobacteria*	*Pseudomonadaceae*	*Pseudomonas*	negative
**PD2**	root71	*Proteobacteria*	*Gammaproteobacteria*	*Pseudomonadaceae*	*Pseudomonas*	negative
***PS**	WCS417	*Proteobacteria*	*Gammaproteobacteria*	*Pseudomonadaceae*	*Pseudomonas (simiae)*	negative
***PP**	WCS358	*Proteobacteria*	*Gammaproteobacteria*	*Pseudomonadaceae*	*Pseudomonas (putida)*	negative
***AC**	root1280	*Proteobacteria*	*Gammaproteobacteria*	*Moraxellaceae*	*Acinetobacter*	negative
**PX**	root630	*Proteobacteria*	*Gammaproteobacteria*	*Xanthomonadaceae*	*Pseudoxanthomonas*	negative
***AH1**	root170	*Proteobacteria*	*Betaproteobacteria*	*Alcaligenaceae*	*Achromobacter*	negative
**AH2**	root83	*Proteobacteria*	*Betaproteobacteria*	*Alcaligenaceae*	*Achromobacter*	negative
***AD1**	root70	*Proteobacteria*	*Betaproteobacteria*	*Comamonadaceae*	*Acidovorax*	negative
**AD2**	root217	*Proteobacteria*	*Betaproteobacteria*	*Comamonadaceae*	*Acidovorax*	negative
***VA1**	root318D1	*Proteobacteria*	*Betaproteobacteria*	*Comamonadaceae*	*Variovorax*	negative
**VA2**	root473	*Proteobacteria*	*Betaproteobacteria*	*Comamonadaceae*	*Variovorax*	negative
***SP1**	root241	*Proteobacteria*	*Alphaproteobacteria*	*Sphingomonadaceae*	*Sphingomonas*	negative
**SP2**	root1294	*Proteobacteria*	*Alphaproteobacteria*	*Sphingomonadaceae*	*Sphingomonas*	negative
**SP3**	root1497	*Proteobacteria*	*Alphaproteobacteria*	*Sphingomonadaceae*	*Sphingopyxis*	negative
**RH1**	root149	*Proteobacteria*	*Alphaproteobacteria*	*Rhizobiaceae*	*Rhizobium*	negative
**RH2**	root1203	*Proteobacteria*	*Alphaproteobacteria*	*Rhizobiaceae*	*Rhizobium*	negative
***AG1**	root1240	*Proteobacteria*	*Alphaproteobacteria*	*Rhizobiaceae*	*Agrobacterium*	negative
**AG2**	Root491	*Proteobacteria*	*Alphaproteobacteria*	*Rhizobiaceae*	*Agrobacterium*	negative
**RH3**	root1298	*Proteobacteria*	*Alphaproteobacteria*	*Rhizobiaceae*	*Rhizobium*	negative
**RH4**	root483D2	*Proteobacteria*	*Alphaproteobacteria*	*Rhizobiaceae*	*Rhizobium*	negative
**RH5**	root1204	*Proteobacteria*	*Alphaproteobacteria*	*Rhizobiaceae*	*Rhizobium*	negative
**RH6**	root482	*Proteobacteria*	*Alphaproteobacteria*	*Rhizobiaceae*	*Rhizobium*	negative
**RH7**	root1212	*Proteobacteria*	*Alphaproteobacteria*	*Rhizobiaceae*	*Rhizobium*	negative
**MY**	root265	*Actinobacteria*	*Actinobacteria*	*Mycobacteriaceae*	*Mycobacterium*	positive
**AR**	root4	*Actinobacteria*	*Actinobacteria*	*Microbacteriaceae*	*Agromyces*	positive
**MI**	root166	*Actinobacteria*	*Actinobacteria*	*Microbacteriaceae*	*Microbacterium*	positive
***PA**	root444D2	*Firmicutes*	*Bacilli*	*Paenibacillaceae*	*Paenibacillus*	positive
***FL1**	Root186	*Bacteroidetes*	*Flavobacteriia*	*Flavobacteriaceae*	*Flavobacterium*	negative
***FL2**	root901	*Bacteroidetes*	*Flavobacteriia*	*Flavobacteriaceae*	*Flavobacterium*	negative

Abb.: abbreviation of strains

*Strains used for accuracy evaluation (Fig. 2c) and for SynCom12 and SynCom12_unequal (Fig. 3 and Fig. 4).

All 30 strains were used for SynCom30 (Fig. 3).

Supplementary Table 3. Information of probes used in this study.

Oligonucleotide probes were designed to target bacterial 16S rRNA or 23S rRNA (see Probe design in Methods). rRNA sequences of PP and PS were downloaded from NCBI. rRNA sequences of other bacterial strains used in this study were extracted from whole genome sequencing data using Prokka. The whole genome sequences of root isolates were acquired from http://www.at-sphere.com/. Source data are provided as a Source Data file.

All targeted probes designed in this study were conjugated to 3 types of fluorophores (FAM, Cy3 and Cy5). The universal probe (EUB338) was conjugated to Cy5.

Information of probes used in SynCom12 and SynCom30 (designed by the custom pipeline in Supplementary Fig. 4).

Abb. of target taxa	Target strain	Probe Sequence 5'-3'	Target Region	ΔG^a (KJ/mol)	FA %[b]	16S MM[c]	23S MM[d]	WGS MM[e]
**PD1**	root401	GTCCTACTCGATTTCACTTC	23S	-16.3	31.1	7	3	3
**PD2**	root71	TCCGCCGCTGAATTCAGGAGC	16S	-14	35.7	3	7	3
**PS**	WCS417	TTAGGTAACTGCCCTTCCTC	16S	-14.7	34.7	2	7	2
**PP**	WCS358	CTGTGTCAGAGTTCCCGAAG	16S	-15.3	30.8	3	7	3
**AC**	root1280	ACAAGTGATCCCCTGCTTTCC	16S	-16	39.9	4	5	3
**PX**	root630	CATCTAATCGCGTGAGGCCTT	16S	-16.6	37	6	7	3
**AH1**	root170	CCGGAACGTTTCTTTCCTGCC	16S	-15.1	40.1	3	6	3
**AH2**	root83	CCATGACGTTTCTTTCCTGCC	16S	-14.8	34.4	3	7	3
**AD1**	root70	CCCAGGTATTATCCAGAGTC	16S	-16.1	34.7	5	6	3
**AD2**	root217	GTCATGGACCCCCTTTATT	16S	-14.2	30.4	4	6	3
**VA1**	root318D1	TACCTTTCGGTGGGTTTCCC	23S	-15.8	43	5	4	3
**VA2**	root473	GGTCGTTGTTAGCTGAAGCT	23S	-16.1	35.6	6	3	3
**SP1**	root241	TGGTCTTTCGACATCATCCGG	16S	-15.4	33.3	4	6	3
**SP2**	root1294	TCAACAGTCGTCCAGTGAGC	16S	-17.7	38.2	4	6	3
**SP3**	root1497	TACTTGTCCAGTCAGTCGC	16S	-17.4	39	4	5	3
**RH1**	root149	TCACACTCGCGTGCTCGCTG	16S	-13.2	35.4	5	6	3
**RH2**	root1203	ATCTCTGCAAGTAGCCGGGC	16S	-14.2	34.3	4	7	3
**AG1**	root1240	TCTCCGG TAACCGCGACCCA	16S	-14.9	44.5	6	5	3
**AG2**	Root491	ACCCCGAATGTCAAGAGCTG	16S	-14.7	27.9	4	6	3
**RH3**	root1298	AACGTCTCCGTAATCCGCGA	16S	-14.1	30.9	3	7	3
**RH4**	root483D2	GCCGCTCGTATTGCTACGC	16S	-14.3	37.3	5	4	2
**RH5**	root1204	CATTACTGCGTATCCTCAGCT	23S	-14.6	29.9	7	3	3
**RH6**	root482	TTGCTCATGTATCCTCAGCT	23S	-15.4	32.9	5	5	2
**RH7**	root1212	ACCTCTCGGTCGTATACGGTA	16S	-14.4	31.2	4	6	3
**MY**	root265	GGCGCATGGTCATATTCGGT	16S	-16.7	38.3	4	6	3

**AR**	root4	AGATGCCTCCGAGGGTCGT	16S	-13.3	32.5	5	7	3
**MI**	root166	GCGGTCACGTCTCGTATCCA	16S	-14.2	36.3	5	7	3
**PA**	root444D2	GGCCCATCTATAAGCCACAGA	16S	-14.9	35.6	5	6	3
**FL1**	Root186	ACCGTCAAGTCCCGACACGT	16S	-16	39.6	6	7	3
**FL2**	root901	GCGAGGTGGCTGCTCTCTGT	16S	-15.2	43.6	3	7	3
Universal	All	GCTGCCTCCCGTAGGAGT	16S	N/A	N/A	0	N/A	N/A

[a] ΔG, the overall Gibbs free energy change for probe-target hybridization calculated by mathFISH.
[b] FA%, melting formamide concentration calculated by mathFISH.
[c] 16S MM, the minimal mismatch between the probe sequence and the 16S rRNA sequence of non-target strains.
Probes have at least three central mismatches to 16S rRNA sequences of non-target taxa, except the probe of PS (only two mismatches to PD1 and PD2).
[d] 23S MM, the minimal mismatch between the probe sequence and the 23S rRNA sequence of non-target strains.
[e] WGS MM, the minimal mismatch between the probe sequence and all mRNA sequences of non-target strains.

Information of probes used in 130 strains (designed by DECIPHER). The efficiency and melting formamide concentration (FA%) of probes are given by DECIPHER. Strains are also isolated from *Arabidopsis* root microbiota, and detailed information can be acquired from http://www.at-sphere.com/.

**No.**	**Strain**	**Target**	**Probe Sequence 5'-3'**	**Efficiency**	**FA %**
1	Root217	Target1	GTCCGCGCAAGGCCTTGC	0.8639	47.2569
2	Root219				
3	Root267				
4	Root275				
5	Root568	Target2	CCACTCTCGGGCACGTTCC	0.8544	45.8703
6	Root70				
7	Root627	Target3	GCATCTCTGCTGGATTCCGGA	0.841	43.8446
8	Root480				
9	Root65	Target4	GTGTTGGCCCAGGATGCC	0.8382	45.3654
10	Root630				
11	Root483D1	Target5	GCGCCACTGAAGTGCATGCAC	0.8103	42.0684
12	Root381				
13	Root553	Target6	GCCCATCCCCAACCAATAAATC	0.7518	38.8863
14	Root280D1				
15	Root68	Target7	CCGCCGCTGAATTCAGGAGCA	0.8542	44.6933
16	Root71				
17	Root569	Target8	TCTACAACCCTTCCTCCC	0.7142	38.5521
18	Root562	Target9	GCAAGGTATTCGCTTACTGCCC	0.8467	43.703
19	Root100	Target10	CCCCTAACTTAACAATCCGCCT	0.7855	40.4069
20	Root102				
21	Root695				
22	Root604	Target11	GTCATTCCCCCCGAGTATT	0.8122	43.1375

23	Root916				
24	Soil810	Target12	GCCACTGATCAGACCAAGCAAGC	0.8081	41.1322
25	Root318D1	Target13	CCACTCGCCGCCAGGATT	0.822	44.2911
26	Root473				
27	Root411				
28	Root434				
29	Root1252	Target14	ACATCTCTGTAATCCGCGATCGG	0.7101	36.9389
30	Root127				
31	Root231				
32	Root258				
33	Root172	Target15	CCAACGGCTAGCTTCCATCGT	0.8306	43.2168
34	Root1433	Target16	GCGGTCACGTCACATATCCAGT	0.8311	42.7741
35	Root137	Target17	CAGACCAGCAAGCTGGCCA	0.7579	40.2353
36	Root930				
37	Root401	Target18	CCGCTCTCAAGAGGTGCAAGCA	0.8389	43.2282
38	Root483D2	Target19	CCGCTCGTATTGCTACGCGC	0.833	43.8635
39	Root1497	Target20	CCAAGCTCTATGAGCCCGGAC	0.8309	43.231
40	Soil745	Target21	CTTCTTCGTTCACGCGGCGT	0.7947	41.6791
41	Root170	Target22	TTCCGAACCGCCTGCGCA	0.8066	43.3371
42	Root565				
43	Root83				
44	Root1280	Target23	CAGGAGCCTCCTCCTCGC	0.8865	49.1968
45	Root472D3	Target24	CCCAACATGCGAAGGGGGTTCA	0.8273	42.5607
46	Root123D2	Target25	GTGCTCCAGGCTCCGAAGAG	0.8078	42.3901
47	Root491	Target26	GCTATTCCGCAGGGCACGG	0.8143	43.2606
48	Root651	Target27	GGCCAATCCTTCCCCGAT	0.8346	45.1167
49	Root81	Target28	CCGAAGCCCTTCTTCCCT	0.8426	45.6698
50	Root1464	Target29	CCCAGGTGCAAGCACCCG	0.8424	45.6537
51	Soil535	Target30	CCTATTTCTAGGGCGTTCCC	0.806	42.2864
52	Soil762	Target31	CCTGTTTCCAGGTATTTCCGG	0.8012	41.5834
53	Soil761	Target32	CCACCTGTAAACCAGCCCC	0.8391	44.8155
54	Soil764	Target33	ACCTTGCGGCTTCTTCCC	0.8259	44.5398
55	Root147	Target34	GGGGAACGCTCTATCTCTAGAGT	0.7427	38.2084
56	Root239				
57	Root11	Target35	CCGTCAAGGTGCCAGCTTATTC	0.8343	42.9599
58	Root131				
59	Root9	Target36	CTTTCATCCTTGAACCATGCGGT	0.7905	40.2816
60	Root920				
61	Root670	Target37	CCACTGAAGAGCAAGCTCCCC	0.8482	44.301
62	Root186	Target38	CCGTCAAGTCCCGACACGTCG	0.8539	44.6718
63	Root901	Target39	GCGAGGTGGCTGCTCTCTG	0.816	43.3609
64	Root935	Target40	CATCCCGATGCCGGGTCG	0.8077	43.4013
65	Root420	Target41	GCTTCATGCGAAGCTGCTATGCT	0.8488	43.3585
66	Root209	Target42	GCACTCCAGCTTTGCAGTCA	0.8151	42.7975
67	Root107	Target43	CCAACCCCCATGCAGAGGA	0.8291	44.1689

68	Root187				
69	Soil729	Target44	GTTCGCCACTGCTCACCC	0.8498	46.1829
70	Root227	Target45	GCCTCCGTACGTCATATCCG	0.811	42.5677
71	Root60	Target46	GCCTTCGCAGCTCGTATCC	0.8506	45.601
72	Root690	Target47	CATGATCCCCGGGTATTAACCG	0.7649	39.4574
73	Root224	Target48	GCCCAGAGTTAAGCCCTGG	0.8219	43.7148
74	Root236	Target49	TCCACCTCAGAACATGTGATCCG	0.7616	38.9924
75	Root685	Target50	CCATCCAACGGCTAGCTCTCA	0.8348	43.4665
76	Root166	Target51	GCGGTCACGTCTCGTATCCA	0.8166	42.8862
77	Root53	Target52	GCGGGGGGACTGTTTCCAG	0.8006	42.4678
78	Root4	Target53	GCCCCCAGATTTCACAGCA	0.8473	45.3699
79	Root135	Target54	GCACGCCCACAGTTGAGCT	0.8317	44.3307
80	Root265	Target55	CATCTCTGCCGGCGTCCT	0.8523	46.3673
81	Root136	Target56	GCTTCGTCCCTGTCAAAAGCGG	0.8285	42.6295
82	Root190	Target57	GCGTGAAGTGGTCCTATCCGG	0.8275	43.0314
83	Root79	Target58	CCGCTGTTTTCACTCCCGAC	0.8306	43.7155
84	Root614	Target59	CCCCATGCAGGGACATGTCA	0.8449	44.6263
85	Root140	Target60	CCACAACCACCAGATGCCTGG	0.8369	43.5947
86	Root151				
87	Soil777	Target61	CCACCCACAACCATGCAGTCAA	0.8334	42.9047
88	Soil805	Target62	CCCCAACCCATGCGGATCG	0.8481	45.4235
89	Root22	Target63	CCAGACACTCCCCATGCAG	0.7884	41.798
90	Root918				
91	Root444D2	Target64	CCTTGCTGTTCGTCTAGGGC	0.8265	43.4713
92	Root52	Target65	CCGTCACCTCAGGAGCAGTT	0.8303	43.7008
93	Root101	Target66	GCCGCGTTCTCGCATATGTCAA	0.8447	43.5791
94	Soil748				
95	Soil803	Target67	CCAGACGATAGCCATGCAGCTT	0.8247	42.4163
96	Root1277	Target68	CCAGATCTCTCTGGTGGTCC	0.8226	43.2344
97	Root1290				
98	Root700				
99	Soil756	Target69	CCCATCTCTGAGCGTTTCCG	0.8279	43.5526
100	Root708	Target70	GCAGCTGTCCGGGCATGT	0.8342	45.0929
101	Root1204	Target71	GTAGAGTTGGGTATGTTCCCAC	0.7522	38.9005
102	Root1203	Target72	GCATCTCTGCAAGTAGCCGGG	0.8426	43.9445
103	Root149	Target73	CTGTGGCGAGCCGGGCAT	0.8316	44.9164
104	Root1240	Target74	GCACCTGTCTTGGGTCCAG	0.8212	43.6765
105	Root274				
106	Root1212	Target75	GCGGGCCAATCCATCACC	0.7766	41.638
107	Root482	Target76	CGTCACCGTCTCGCTGCC	0.8513	46.2951
108	Soil772	Target77	CCCGCATCTCTGCAGGATTC	0.8145	42.7677
109	Root241	Target78	CCTCAGCGTCAATACCAGTCC	0.814	42.2681
110	Root710	Target79	CCTCGCGAGTTCGCTGCC	0.8487	46.1027
111	Root1294	Target80	TCGGTGAGTTATTCCGTACCC	0.7585	39.5148
112	Root50				

113	Root720				
114	Root369	Target81	CCACCACAGATGCCTGCGG	0.8211	43.6685
115	Root1319	Target82	GCCCCATGAGGGACAGAGTG	0.8149	42.7899
116	Root264	Target83	GGCGACCATCTCTGGCCG	0.8148	43.8342
117	Root1310	Target84	CCACAAGGGGGCGCCTGT	0.8424	45.6538
118	Root1304	Target85	CCCATGCAGGAGCCGGTG	0.8305	44.8443
119	Root66D1				
120	Soil811	Target86	CCATACCCATGCAGGTTGGT	0.8081	42.4049
121	Soil809	Target87	GCGGGGCGTACATTTCTGCA	0.8015	42.042
122	Root495	Target88	CTTTCCACCTCCGATCATGC	0.7597	39.9316
123	Root179	Target89	CCTCGATCTCTCAAGGATTCCGG	0.829	42.222
124	Root561				
125	Root1298	Target90	GTCTCCGTAATCCGCGATCGG	0.8075	41.9185
126	Root1312				
127	Root278				
128	Root423				
129	Root558				
130	Root74				

Supplementary Table 4. Microscope setting used in this study.

TD: transmitted detector. PMT: photomultiplier tube. HV: high voltage.

**Channel Setting**	**Excitation Wavelength**	**Laser Power**	**PMT HV**	**PMT Offset**	**Emission Wavelength**	**Filter Cube**	**Scan Mode**	**Scan Speed (µs/pixel)**
FAM	488 nm	9 mW	90	0	525 nm	525/50	Channel series	0.5
Cy3	561 nm	3.2 mW	55	0	595 nm	595/50	Channel series	0.5
Cy5	640 nm	16 mW	120	0	700 nm	700/50	Channel series	0.5
TD	N/A	N/A	152	0	N/A	N/A	Channel series	0.5

Supplementary Table 5. Codebooks used in this study.

Color code equals 1, 2 and 3 for FAM, Cy3 and Cy5 fluorophores, respectively.

R=8, HD≥4, S=12, F=3 (Fig. 2 to 5 and Supplementary Fig. 9 to 11)

Strain	Hybridization Rounds							
	**1**	**2**	**3**	**4**	**5**	**6**	**7**	**8**
**PD1**	1	2	1	2	1	1	3	3
**PS**	2	2	2	2	2	2	2	2
**PP**	3	3	2	3	3	3	3	3
**AC**	2	1	1	2	1	2	2	2
**AH1**	3	1	3	1	1	3	2	2
**VA1**	1	3	1	3	1	3	2	1
**AD1**	2	1	2	1	1	2	2	1
**SP1**	2	3	2	3	2	3	1	1
**AG1**	3	3	3	2	2	2	2	1
**PA**	1	2	3	3	2	2	3	2
**FL1**	1	2	3	1	1	3	3	1
**FL2**	1	2	2	1	2	1	2	1

R=8, HD≥4, S=30, F=3 (Fig. 3)

Strain	Hybridization Rounds							
	1	2	3	4	5	6	7	8
PD1	1	3	2	1	3	2	2	3
PD2	1	1	1	1	3	3	3	3
PS	2	1	3	1	1	1	3	3
PP	1	1	3	2	3	1	3	1
AC	3	3	3	2	2	2	2	1
PX	2	2	2	3	2	1	3	3
AH1	1	3	1	3	3	1	3	2
AH2	1	2	3	3	2	2	3	2
AD1	2	2	2	2	2	2	2	2
AD2	2	1	1	2	1	2	1	2
VA1	3	1	2	3	3	1	2	1
VA2	3	2	1	3	1	2	2	3
SP1	1	2	1	2	2	3	1	2
SP2	2	2	2	2	1	1	1	1
SP3	3	2	3	3	2	1	1	1
RH1	3	1	2	1	3	2	3	2
RH2	1	2	3	1	3	1	1	3
AG1	3	1	3	1	1	3	2	2
AG2	2	3	1	1	1	3	1	3
RH3	1	1	2	2	1	1	2	2
RH4	1	1	2	3	3	3	1	2
RH5	1	2	2	1	1	2	1	2
RH6	2	3	1	2	3	3	3	1
RH7	3	1	2	2	1	3	3	1
MY	3	3	2	3	3	3	3	3
AR	2	2	1	1	1	1	2	2
MI	2	2	3	1	2	3	2	3
PA	1	3	1	3	1	3	2	1
FL1	2	1	1	3	3	1	1	3
FL2	2	1	1	3	2	3	3	2

R=12, HD≥6, S=30, F=3 (Fig. 3)

Strain	Hybridization Rounds											
	**1**	**2**	**3**	**4**	**5**	**6**	**7**	**8**	**9**	**10**	**11**	**12**
**PD1**	1	2	1	1	1	3	3	1	1	2	3	2
**PD2**	1	2	1	1	2	1	2	3	1	2	1	3
**PS**	2	1	1	1	2	3	3	2	2	3	2	3
**PP**	1	2	2	1	2	3	1	3	1	3	2	1
**AC**	3	3	3	3	3	2	1	1	1	3	2	3
**PX**	2	2	2	1	3	2	2	2	1	3	3	1
**AH1**	2	3	2	2	1	2	3	3	3	1	1	1
**AH2**	3	1	2	3	2	2	1	1	3	1	1	1
**AD1**	2	1	2	2	3	2	1	2	3	2	2	1
**AD2**	1	2	2	3	3	3	2	1	2	2	3	1
**VA1**	2	1	3	1	1	1	2	2	1	3	1	3
**VA2**	2	1	3	2	3	3	2	1	3	3	1	1
**SP1**	1	1	1	2	2	1	3	1	3	2	2	1
**SP2**	2	2	1	2	1	1	2	3	1	1	3	1
**SP3**	1	2	1	1	1	2	3	2	2	1	1	3
**RH1**	1	1	1	2	2	1	1	3	2	3	1	2
**RH2**	2	1	1	1	2	3	2	3	3	2	3	2
**AG1**	2	2	2	1	1	3	2	1	3	1	1	2
**AG2**	2	2	1	3	1	3	3	3	3	1	2	3
**RH3**	1	2	3	2	1	3	3	3	3	3	1	2
**RH4**	3	2	2	1	1	1	1	2	3	3	3	2
**RH5**	1	2	2	2	3	1	1	3	1	2	3	2
**RH6**	3	1	3	2	2	2	3	1	2	3	1	3
**RH7**	3	1	2	3	1	1	2	3	3	2	2	3
**MY**	1	2	2	2	2	2	3	2	2	2	2	2
**AR**	1	1	3	3	2	1	2	2	2	1	3	1
**MI**	2	2	1	2	3	2	1	2	1	1	1	2
**PA**	2	3	3	1	1	3	3	2	2	2	3	1
**FL1**	2	1	3	1	2	1	1	3	2	2	2	1
**FL2**	1	1	2	2	1	3	1	3	2	1	3	3

R=9, HD≥4, S=130, F=3 (Supplementary Fig. 19)

Taxa	Hybridization Rounds								
	1	2	3	4	5	6	7	8	9
Target1	1	1	2	3	3	2	2	3	2
Target2	3	3	1	3	2	3	2	2	3
Target3	3	1	2	1	3	2	1	3	1
Target4	1	1	3	1	3	1	3	2	1
Target5	2	1	2	1	3	1	1	1	2
Target6	3	1	1	3	3	1	2	2	1
Target7	1	2	3	1	1	1	3	1	3
Target8	2	1	3	2	1	1	1	3	3
Target9	1	2	1	3	1	1	1	3	3
Target10	1	2	1	2	1	1	2	2	1
Target11	3	1	3	1	1	1	2	3	1
Target12	1	1	1	2	3	3	3	1	2
Target13	3	3	3	3	3	2	2	3	3
Target14	2	2	3	3	2	1	1	2	2
Target15	2	3	1	1	3	2	2	1	2
Target16	1	1	1	3	2	3	1	3	1
Target17	1	2	3	2	3	2	3	1	1
Target18	2	3	2	1	2	1	3	2	1
Target19	2	1	1	3	1	1	2	3	2
Target20	3	1	1	3	1	3	1	2	2
Target21	1	1	1	2	3	1	2	3	3
Target22	1	2	2	1	1	2	2	1	1
Target23	2	1	1	3	3	3	2	1	3
Target24	1	2	2	3	1	2	3	2	3
Target25	1	2	2	2	2	2	2	2	2
Target26	3	3	3	1	2	3	1	2	1
Target27	1	2	2	2	3	2	1	3	3
Target28	2	2	3	1	1	3	2	1	2
Target29	2	3	1	1	1	3	2	2	1
Target30	2	2	2	1	3	2	3	1	3
Target31	1	2	3	3	2	2	1	1	3
Target32	2	1	1	1	2	3	3	1	1
Target33	3	3	3	3	1	3	3	3	1
Target34	1	1	1	2	2	2	2	1	1
Target35	1	3	2	1	1	3	1	3	1
Target36	1	2	2	3	3	1	3	1	2
Target37	3	3	2	2	1	2	1	1	3
Target38	3	3	2	1	1	1	3	3	3
Target39	1	3	1	3	1	1	3	2	2
Target40	2	1	2	2	2	3	1	2	3
Target41	1	1	1	1	1	1	1	1	1
Target42	2	3	2	2	2	2	3	3	2

**Target43**	3	2	1	3	1	2	2	3	1
**Target44**	1	3	1	2	2	1	3	3	1
**Target45**	1	2	1	3	3	3	2	2	2
**Target46**	1	3	1	1	2	2	2	3	3
**Target47**	1	2	2	1	1	1	1	2	2
**Target48**	2	1	2	1	1	3	3	2	2
**Target49**	3	2	2	3	2	3	3	2	1
**Target50**	2	1	1	1	2	2	1	3	2
**Target51**	3	2	3	2	3	1	2	1	2
**Target52**	3	2	3	1	3	3	3	2	2
**Target53**	3	1	2	2	1	3	3	1	1
**Target54**	2	2	2	3	1	1	2	1	3
**Target55**	1	3	2	1	3	1	2	2	3
**Target56**	2	3	1	2	2	3	1	1	2
**Target57**	1	1	3	1	3	3	1	3	2
**Target58**	3	2	1	3	3	3	3	3	3
**Target59**	2	1	2	3	3	1	3	2	3
**Target60**	1	1	3	2	1	2	3	3	2
**Target61**	3	3	1	3	3	1	1	3	2
**Target62**	2	1	2	1	1	2	2	3	3
**Target63**	2	1	3	3	1	2	1	1	1
**Target64**	3	2	1	1	3	2	2	2	3
**Target65**	3	2	3	1	1	2	1	3	2
**Target66**	3	1	2	3	2	2	2	1	3
**Target67**	1	1	2	1	2	1	1	3	3
**Target68**	1	3	1	2	3	3	1	2	1
**Target69**	1	2	3	3	3	1	2	3	1
**Target70**	3	2	1	2	1	3	2	1	3
**Target71**	2	2	1	2	3	3	2	3	1
**Target72**	2	3	3	3	3	3	3	1	2
**Target73**	2	2	2	3	2	2	1	3	1
**Target74**	1	2	1	1	2	1	2	1	2
**Target75**	1	1	2	3	1	3	1	1	3
**Target76**	1	1	1	2	2	1	1	2	2
**Target77**	1	1	1	3	2	1	3	1	3
**Target78**	1	2	1	2	2	3	3	2	3
**Target79**	1	2	2	3	2	3	2	3	3
**Target80**	1	2	2	2	2	1	1	1	1
**Target81**	3	2	2	3	3	2	1	2	2
**Target82**	1	2	1	1	3	2	3	3	2
**Target83**	2	3	2	1	2	3	2	1	3
**Target84**	2	1	1	1	2	1	2	2	3
**Target85**	2	2	1	1	1	1	3	3	1
**Target86**	3	1	3	2	3	3	1	1	3
**Target87**	2	2	1	2	3	1	1	2	3

**Target88**	3	3	2	3	1	1	1	2	1
**Target89**	1	1	1	1	1	3	3	3	3
**Target90**	1	3	3	2	3	3	3	3	3

SynCom30.2 (30 strains) is a subset of 130 strains. The information of probes can be found in Supplementary Table 3.

R=8, HD⩾4, S=30, F=3 (Fig. 6)

Strain ID	Name	Target	Hybridization Rounds							
			1	2	3	4	5	6	7	8
**Root217**	*Acidovorax* sp.	**Target1**	1	1	2	3	1	2	3	3
**Root68**	*Pseudomonas* sp.1	**Target7**	3	1	3	1	1	3	2	2
**Root569**	*Pseudomonas* sp.2	**Target8**	1	2	3	1	3	1	1	3
**Root473**	*Variovorax* sp.	**Target13**	3	2	3	3	2	1	1	1
**Root231**	*Sinorhizobium* sp.1	**Target14**	2	1	1	2	2	1	2	1
**Root172**	*Mesorhizobium* sp.	**Target15**	2	3	3	3	1	2	3	1
**Root930**	*Cellulomonas* sp.	**Target17**	3	3	1	1	2	1	2	3
**Root483D2**	*Rhizobium* sp.1	**Target19**	1	1	2	2	1	1	2	2
**Soil745**	*Bacillus* sp.3	**Target21**	3	1	2	3	3	1	2	1
**Root170**	*Achromobacter* sp.	**Target22**	1	1	2	2	2	2	1	1
**Root1280**	*Acinetobacter* sp.	**Target23**	2	2	3	3	1	3	1	2
**Root491**	*Agrobacterium* sp.	**Target26**	1	3	1	3	3	1	3	2
**Root81**	*Agromyces* sp.2	**Target28**	3	2	1	3	1	2	2	3
**Root1464**	*Agromyces* sp.1	**Target29**	2	2	1	1	1	1	2	2
**Soil762**	*Arthrobacter* sp.2	**Target31**	3	1	2	2	1	3	3	1
**Soil764**	*Arthrobacter* sp.1	**Target33**	2	1	2	2	3	3	2	3
**Root147**	*Bacillus* sp.1	**Target34**	1	2	1	2	3	2	2	1
**Root131**	*Bacillus* sp.2	**Target35**	1	1	3	3	2	1	2	3
**Root935**	*Flavobacterium* sp.	**Target40**	2	2	3	1	2	3	2	3
**Root690**	Lysobacter sp.	**Target47**	3	2	1	1	3	1	3	1
**Root79**	*Nocardioides* sp.1	**Target58**	2	3	1	1	1	3	1	3
**Root151**	*Nocardioides* sp.2	**Target60**	3	3	2	2	1	2	1	3
**Root22**	*Oerskovia* sp.	**Target63**	3	3	3	2	2	2	2	1
**Root444D2**	*Paenibacillus* sp.1	**Target64**	2	3	2	3	2	3	1	1
**Root52**	*Paenibacillus* sp.2	**Target65**	2	3	1	2	3	3	3	1
**Root708**	*Rhizobium* sp.2	**Target70**	2	2	2	3	2	1	3	3
**Root149**	*Rhizobium* sp.3	**Target73**	1	2	2	1	1	2	1	2
**Root50**	*Sphingomonas* sp.	**Target80**	1	3	1	3	2	2	1	3
**Root179**	*Rhodanobacter* sp.	**Target89**	1	1	1	1	2	2	2	2
**Root1312**	*Sinorhizobium* sp.2	**Target90**	2	3	3	2	2	1	1	3

Codebook used for probe specificity analysis (Supplementary Fig. 6)

Strain	Hybridization Rounds											
	1	2	3	4	5	6	7	8	9	10	11	12
PD1	1	0	0	0	2	0	0	0	3	0	0	0
PS	2	0	0	0	3	0	0	0	1	0	0	0
PP	3	0	0	0	1	0	0	0	2	0	0	0
AC	0	1	0	0	0	2	0	0	0	3	0	0
AH1	0	2	0	0	0	3	0	0	0	1	0	0
VA1	0	3	0	0	0	1	0	0	0	2	0	0
AD1	0	0	1	0	0	0	2	0	0	0	3	0
SP1	0	0	2	0	0	0	3	0	0	0	1	0
AG1	0	0	3	0	0	0	1	0	0	0	2	0
PA	0	0	0	1	0	0	0	2	0	0	0	3
FL1	0	0	0	2	0	0	0	3	0	0	0	1
FL2	0	0	0	3	0	0	0	1	0	0	0	2
