# Supplementary material for: Spatial profiling of microbial communities by sequential FISH with error-robust encoding
Source: Nat Commun. 2023 Mar 17;14:1477. doi: 10.1038/s41467-023-37188-3 (PMC10023729; doi:10.1038/s41467-023-37188-3)
Supplement: Supplementary file 2 — Reporting Summary [file 41467_2023_37188_MOESM2_ESM.pdf]

nature portfolio

Corresponding author(s): Lei Dai

Last updated by author(s): Feb 28, 2023

Reporting Summary

Nature Portfolio wishes to improve the reproducibility of the work that we publish. This form provides structure for consistency and transparency in reporting. For further information on Nature Portfolio policies, see our Editorial Policies and the Editorial Policy Checklist.

Statistics

For all statistical analyses, confirm that the following items are present in the figure legend, table legend, main text, or Methods section.

n/a	Confirmed	
☐	☒	The exact sample size (n) for each experimental group/condition, given as a discrete number and unit of measurement
☐	☒	A statement on whether measurements were taken from distinct samples or whether the same sample was measured repeatedly
☐	☒	The statistical test(s) used AND whether they are one- or two-sided<br>*Only common tests should be described solely by name; describe more complex techniques in the Methods section.*
☒	☐	A description of all covariates tested
☐	☒	A description of any assumptions or corrections, such as tests of normality and adjustment for multiple comparisons
☐	☒	A full description of the statistical parameters including central tendency (e.g. means) or other basic estimates (e.g. regression coefficient) AND variation (e.g. standard deviation) or associated estimates of uncertainty (e.g. confidence intervals)
☐	☒	For null hypothesis testing, the test statistic (e.g. F, t, r) with confidence intervals, effect sizes, degrees of freedom and P value noted<br>*Give P values as exact values whenever suitable.*
☒	☐	For Bayesian analysis, information on the choice of priors and Markov chain Monte Carlo settings
☒	☐	For hierarchical and complex designs, identification of the appropriate level for tests and full reporting of outcomes
☐	☒	Estimates of effect sizes (e.g. Cohen's d, Pearson's r), indicating how they were calculated

Our web collection on statistics for biologists contains articles on many of the points above.

Software and code

Policy information about availability of computer code

Data collection	Imaging data were acquired using the NIS-Elements AR 4.60.00. Illumina sequencing data were collected with MiSeq Control Software v2.6.2.1.
Data analysis	All image analysis code are written in Matlab 2022b. Sequencing data analysis code are written in Python (v3.7.12). In addition, following tools and algorithms were used, Prokka (v1.14.6), ARB (v6.0.6), mathFISH (http://mathfish.cee.wisc.edu/), and DECIPHER (v2.18.1). See the Methods section for the detail usages. All code are available at https://github.com/JacobZuo/SEER-FISH.

For manuscripts utilizing custom algorithms or software that are central to the research but not yet described in published literature, software must be made available to editors and reviewers. We strongly encourage code deposition in a community repository (e.g. GitHub). See the Nature Portfolio guidelines for submitting code & software for further information.

Data

Policy information about availability of data

All manuscripts must include a data availability statement. This statement should provide the following information, where applicable:

- Accession codes, unique identifiers, or web links for publicly available datasets
- A description of any restrictions on data availability
- For clinical datasets or third party data, please ensure that the statement adheres to our policy

Data reported in this study are deposited to Zenodo on https://doi.org/10.5281/zenodo.5100490.

Human research participants

Policy information about studies involving human research participants and Sex and Gender in Research.

Reporting on sex and gender	This study did not involve human research participants.
Population characteristics	N/A
Recruitment	N/A
Ethics oversight	N/A

Note that full information on the approval of the study protocol must also be provided in the manuscript.

Field-specific reporting

Please select the one below that is the best fit for your research. If you are not sure, read the appropriate sections before making your selection.

- [x] Life sciences
- [ ] Behavioural & social sciences
- [ ] Ecological, evolutionary & environmental sciences

For a reference copy of the document with all sections, see nature.com/documents/nr-reporting-summary-flat.pdf

Life sciences study design

All studies must disclose on these points even when the disclosure is negative.

Sample size	No statistical method was used to predetermine the sample size. The sample size in this study was based on typical numbers used for imaging investigations. Post hoc analysis on reproducibility indicates that the sample size is sufficient. For representative images and analysis based on a single imaging experiment (figure 2c , supplementary figures 1c,3,19), a sufficient number of bacterial cells were imaged.
Data exclusions	No data were excluded from the analysis.
Replication	For in vivo experiments, three or ten biological replicates were imaged and experimental results were reproducible. For in vitro experiments, high Pearson's correlation coefficient exceeding 0.9 indicates the reproducibility of the method based on thousands of cells from three independent experiments.
Randomization	Allocation of samples was random.
Blinding	Blinding was not applied this study. Image acquisition and analyses were performed following an automated workflow for samples in different experiments or in different groups.

Reporting for specific materials, systems and methods

We require information from authors about some types of materials, experimental systems and methods used in many studies. Here, indicate whether each material, system or method listed is relevant to your study. If you are not sure if a list item applies to your research, read the appropriate section before selecting a response.

Materials & experimental systems

n/a	Involved in the study	
x		Antibodies
x		Eukaryotic cell lines
x		Palaeontology and archaeology
x		Animals and other organisms
x		Clinical data
x		Dual use research of concern

Methods

n/a	Involved in the study	
x		ChIP-seq
x		Flow cytometry
x		MRI-based neuroimaging
